# Phylogenomics of Western Eurasian *Tilia*: merging GBS datasets to place the Hyrcanian forest limes

**DOI:** 10.1186/s12870-025-07435-4

**Published:** 2025-10-14

**Authors:** Nastaran Ala, Ali Bagheri, Habib Zare, Axel Himmelbach, Dörte Harpke, Frank R. Blattner

**Affiliations:** 1 Department of Plant and Animal Biology, Faculty of Biological Science and Technology, University of Isfahan, Isfahan, 81746-73441 Iran; 2 Botanical Garden of Nowshahr, Research Institute of Forests and Rangelands, Agricultural Research, Education and Extension Organization, AREEO, Tehran, Iran; 3 Leibniz Institute of Plant Genetics and Crop Plant Research (IPK), Gatersleben, 06466 Germany; 4 German Centre of Integrative Biodiversity Research (iDiv) Halle-Jena-Leipzig, Leipzig, 04103 Germany

**Keywords:** Evolutionary history, Genotyping-by-sequencing, Iran, Phylogenomic analyses, Ploidy levels, Taxonomy, Tilia

## Abstract

**Background:**

Morphological uniformity in *Tilia* (Malvaceae s.l., Tilioideae) and unclear species delimitations resulted in over 500 names associated with the about 30 species of this genus. This is also an issue for the Hyrcanian Forest diversity hotspot of northern Iran, where between one and seven *Tilia* species were described to occur. We used genotyping-by-sequencing (GBS) to analyze the phylogeny of western Eurasian *Tilia* species, place the Hyrcanian *Tilia* populations, and infer their phylogeography. To arrive at a complete taxon sample, we merged our own data (104 samples) with a *Tilia* GBS dataset available in GenBank (11 samples) and processed and analyzed them together.

**Results:**

We confirmed GBS data to be additive, i.e. independent datasets can be merged. We found three major groups within the Eurasian *Tilia* species and our analyses were able to resolve the phylogenetic relationships of the species. Neither *T. cordata* nor *T. platyphyllos*, both diploids and previously reported for Iran, are present in the region. Instead, the Iranian *Tilia* samples were identified as tetraploid by flow cytometry. Phylogenetic analyses clearly separated all species and found the Iranian samples to belong to *T. dasystyla* subsp. *caucasica*. The Hyrcanian populations we analyzed are genetically rather uniform and include a group of individuals morphologically resembling *T. sabetii*. Their taxonomic status is currently unclear. Within the Hyrcanian populations we found a colonization pattern from the northwestern to the eastern Hyrcanian forests.

**Conclusions:**

Merging published GBS datasets from different labs will allow the study of geographically widespread taxa like *Tilia*, where relevant species or populations might not all be accessible to individual researchers. Based on the samples we analyzed, the different taxa described for Iran belong genetically to *T*. *dasystyla*, with morphological variation that might be attributed to local environmental factors.

**Supplementary Information:**

The online version contains supplementary material available at 10.1186/s12870-025-07435-4.

## Introduction

The Hyrcanian Forest, located around the southern parts of the Caspian Sea and the northern slopes of the Alborz Mountains in Iran, represent one of the most ancient and ecologically significant temperate forest ecosystems in the world [1]. These forests are known for their high biodiversity and the presence of numerous endemic and relic species [2, 3]. As one of the few remnants of the ancient forests that once covered much of the Northern Hemisphere, the Hyrcanian Forest serves as a refugium for many tree species that have disappeared from other parts of Europe and Western Asia [4] and might have been a source for trees re-colonizing higher latitudes in Eurasia after ice-age cold cycles [5–7]. *Tilia* L. (lime, linden, or basswood) trees are an important broadleaved component of this vegetation unit and often dominate the crown layer of the forests up to elevations of more than 1500 m in protected areas where logging is absent [8]. *Tilia* is widespread in the Northern Hemisphere and the trees are easily recognized by their pale, wing-like leaf attached to the peduncle of their cymose inflorescence [9, 10]. During the warm Atlantic period, approximately 7000 to 4000 years before present, *Tilia* dominated the deciduous forests of parts of Eurasia [11], but declined afterwards with cooler temperatures at higher latitudes and was replaced by beech or oaks. The insect-pollinated trees reach heights of 20 to 40 m with stem diameters of 1 to 3 m and ages of well above 500 years [10]. In contrast to the easy recognition of specimens belonging to the genus, determination of taxa within *Tilia* is impeded by the overall high similarity of species with few qualitative characters discerning them. In addition, morphological traits of branches, leaves and fruits are quite variable depending on their position on the tree. Thus, shape, color and indumentum of the organs could be different if growing at the upper and sun-exposed outer parts of a tree in comparison to the ones at lower positions and within the canopy [8, 10]. Moreover, the frequent occurrence of homoploid hybrids [10] and allopolyploid species [12] in the genus contribute to the difficulties of morphological species delimitation. Accordingly, various species numbers are found in the literature, such as 23 [10, 13], 25 [14], and 30 species [15, 16], and more than 500 taxon names are attached to them [17]. These taxonomic challenges highlight the necessity of integrating phylogenomic approaches with comprehensive sampling to resolve unclear species boundaries and evaluate evolutionary relationships within *Tilia*.

Species number estimations of *Tilia* differ widely for the Hyrcanian Forest. Browicz [18] in Flora Iranica considered only *T. platyphyllos* Scop., present with subsp. *platyphyllos* and subsp. *caucasica* (Rupr.) Loria, while Ghorbanalizadeh and Akhani [19] accepted just *T. platyphyllos* subsp. *caucasica* as single native taxon in Iran. *Tilia dasystyla* Steven was already recorded by Pigott and Francis [20] for the Hyrcanian Forest and Pigott [10] lists *T. dasystyla* subsp. *caucasica* (V.Engl.) Pigott as single lime species for this area. An important point here is that *T. platyphyllos* is diploid (2*x*) while *T. dasystyla* is a tetraploid species (4*x*) [13] involving *T. platyphyllos* as one of its parents [12]. Also *T. begoniifolia *Steven was mentioned for the Hyrcanian Forest by Shishkin and Bobrov [21], while Yousefzadeh et al. [22] doubted the occurrence of *T. platyphyllos* and considered *T. begoniifolia* (listed as *T. begonifolia*), *T. caucasica *Rupr., and *T. dasystyla* to be present. Later, Yousefzadeh et al. [23] described with *T. begoniifolia* (listed as *T. begonifolia*), *T. dasystyla*, *T. hyrcana* Tabari & Colagar, and *T. rubra* DC four species for the Hyrcanian Forest. Zare et al. [8], in a careful study of morphological characters and ecological settings, increased the number of species in Iran to six, including *T. cordata* Mill., *T. dasystyla*, *T. begoniifolia* (listed as *T. begonifolia*), *T. sabetii* H.Zare, *T. stellato-pilosa* H.Zare, Amini & Assadi, and the hybrid species *T. × euchlora* C.Koch. This short overview shows that it is currently not known how many *Tilia* species are present in the Hyrcanian Forest and it is also unclear which species occur, as some of the names listed before like *T. begoniifolia* or *T. rubra*, are seen as synonyms of other species [10]. Still, with up to seven species, the Hyrcanian Forest could be a center of diversity for *Tilia.*

As limes are long-lived trees, mutation rates are low and many of the standard molecular markers for phylogenetic analysis cannot easily resolve relationships among species, subspecies or populations. Thus, even sequencing entire plastid genomes for most *Tilia* species found only three main maternal lineages and different polyphyletic chloroplast types for single species [12], which could easily be attributed to incomplete lineage sorting, given the long generation time of the individuals [24]. The nuclear rDNA internal transcribed spacer region (ITS) provides low resolution, partly due to few informative characters [23, 25, own unpublished data], but also due to intra-individual and intra-species variation in the rDNA cistron [26] that can only be resolved by phasing, i.e. separating the different copies before starting an analysis [27]. Thus, phylogenetic analyses based on multilocus nuclear data combined with entire plastid genomes resulted for the first time in a reasonable species phylogeny for *Tilia* [12] although species borders remained partly unclear, as only single to few individuals per species were included. Another approach used genome-wide single nucleotide polymorphisms (SNP) that are able to resolve even closely related taxa in *Tilia* [28] through genotyping-by-sequencing (GBS; [29]), and seems promising for population analyses and species delimitation. GBS is a reduced genomic representation method, that uses two restriction enzymes to cut genomic DNA that is afterwards characterized by next-generation short-read sequencing of the DNA-stretch close to one of the restriction sides [30]. GBS has proven to be able to reliably resolve phylogenetic relationships among closely related species [28, 31, 32] and also phylogeographic patterns among populations within species [32, 33].

In the course of our project on *Tilia* phylogenomics, a GBS dataset from a Russian lab became available [28] that included *Tilia* individuals from four species (*Tilia amurensis* Rupr., *T. begoniifolia*, *T. cordata*, and *T. taquetii* C.K.Schneid.) from Russia and adjacent countries. Fortunately, the same restriction enzymes as ours within the GBS protocol were used. However, the size selection windows during library preparation and sequencing platforms were slightly different (see below). Despite these differences, the compatibility of the restriction enzyme pair allowed us to test whether the datasets could be reliably merged for joint analysis.

In *Tilia* di-, tetra- and octoploid species are reported and the polyploids seem all to have originated through allopolyploidization, combining different parental genomes [12]. Thus, determination of ploidy level can be an additional instrument helping in species identification and understanding the evolution of species [12, 34, 35]. The high number of chromosomes within *Tilia* (*x* = 41) make correct chromosome counts tedious, particularly in the polyploids with chromosome numbers of, e.g., 2*n* = 4*x* = ~ 160. Therefore, we use flow cytometry-based genome size measurements to infer the ploidy levels of the individuals or populations collected [36].

Despite the wealth of morphological and molecular studies of *Tilia*, several questions remain unanswered, particularly concerning the number and identity of species in the Hyrcanian Forest. This study aims to fill these gaps by utilizing genomic tools to provide a clearer understanding of species boundaries and their evolutionary history. In this study, we aim to investigate (i) the phylogenetic relationships among *Tilia* species from Eurasia using GBS. Here we want to understand species relationships and use them to infer which and how many species occur in the Hyrcanian Forest, and if these species might represent potential source populations for post-Pleistocene colonization of central and northern Eurasia. To do so we (ii) intend to test if GBS data from different labs can be merged to reach a more inclusive dataset regarding taxa and geographic regions represented. In addition (iii) we want to analyze biogeographic or phylogeographic patterns of *Tilia* in the Hyrcanian area, as they can provide evidence for origins of the species and their colonization patterns in the broadleaved forests of this important biodiversity hotspot.

## Materials and methods

### Study area

The Hyrcanian Forest extends along the southern coast of the Caspian Sea, covering the northern slopes of the Alborz Mountains in Gilan, Mazandaran, and Golestan provinces of Iran. Elevation ranges from sea level to over 2800 m. The region has a humid temperate climate with annual precipitation from 900 to over 2000 mm and mean annual temperatures between 6 °C and 20 °C depending on altitude [37, 38]. The forest grows on diverse geological substrates including mainly Cenozoic formations, which contribute to varied soil types from alluvial loams to acidic and skeletal soils [39, 40]. Floristically, the forests are dominated by broadleaved deciduous species such as *Fagus orientalis*, *Parrotia persica*, and *Carpinus betulus*, with many endemics and relict taxa in addition [19].

### Plant material and taxon sampling

We included in our study all western Eurasian *Tilia* species complemented by some individuals from central Siberia, the Russian Far East, and North America. For this dataset 19 individuals from natural stands and botanical gardens were collected and included together with 11 samples of the GBS dataset of Shekhovtsov et al. [28], available at NCBI GenBank within the BioProject PRJNA811982, consisting of four *Tilia* species with several subspecies. In Iran, the natural occurrence of *Tilia* is restricted to the Hyrcanian Forest, i.e. the forest stands in the northernmost part of the country bordering the Caspian Sea. In this area 85 *Tilia* individuals were sampled from 23 populations covering the stands of the entire Hyrcanian forests in Iran. Species were determined using published keys [8, 41] to morphologically belong to seven species. Some of the specimens were identified as “cf.” due to incomplete characteristics, such as the absence of flowers together with ripe fruits, or could not be clearly assigned to a single species and alternatives were indicated by “or” (Supplementary Table S1; tables and figures indicated by “S” are available as supplemental online materials attached to this article). Leaf samples were collected, cleaned, packed, and immediately dried and stored in silica gel till DNA extraction. Voucher specimens of the individuals were deposited in the herbaria of the Leibniz Institute of Plant Genetics and Crop Plant Research (GAT), the Bayrische Staatssamlungen München (M), the Universities of Osnabruck (OSBU) or Isfahan (HUI), for the latter with duplicates in the herbarium of the Nowshahr Botanical Garden. Detailed information about the analyzed taxa is provided in Table S1.

### Analysis of genome sizes to determine ploidy level

Genome sizes were assessed using flow cytometry with a Cyflow Space (Sysmec-Partec) involving propidium iodide (PI) as stain and following the method outlined by Bagheri et al. [42]. The pea cultivar *Pisum sativum* L. ‘Viktoria’ (IPK gene bank accession number PIS 630; 10.25642/IPK/GBIS/27322) was used as the internal size standard (2C DNA content = 9.09 pg) in all measurements together with the CyStain PI Absolute P buffer (Partec), as this buffer system resulted in clearer peaks in flow cytometry of the mucilaginous *Tilia* leave tissue. For some *Tilia* individuals, we measured both silica-gel dried and fresh leaves to evaluate the impact of drying on flow cytometric measurements. We found that the results were not identical but comparable, as they differ by < 5% (data not shown) and allow easily determination of ploidy levels. Tests of diverse tissues, i.e. buds, stalks, young and old leaves, did not result in better performance of flow cytometry, as all contain amble amounts of mucus, resulting often in low nuclei counts. Best results were obtained when using wing leaves/bracts, which generally appear to be dryer and containing less slime.

### DNA extraction and genotyping-by-sequencing

Genomic DNA was isolated from silica gel-dried leaves using the innuPREP Plant DNA Kit (Analytik Jena) with about twice the initial amount of extraction buffer and an additional washing step in comparison to the protocol provided by the manufacturer to account for the mucilaginous tissue of the species. To obtain genome-wide SNP data, we implemented a two enzyme GBS method [30], utilizing the restriction enzymes *Pst*I and *Msp*I. Library preparation and individual barcoding were conducted essentially following the protocol of Wendler et al. [43]. Pooled library DNA was size-fractionated (targeted size-range: 400 to 600 bp) using the preparative Blue Pippin electrophoresis system and 2% Agarose Gel Cassettes (Marker V2) according to standard manufacturer’s protocols (Sage Science). Finally, the library was quantified using qPCR essentially as described previously in Mascher et al. [44]. Sequencing of GBS libraries was performed using the Illumina NovaSeq6000 device (SP reagent kit, v1.5 chemistry) with custom Illumina sequencing primer [43], and single read sequencing (122 cycles for read 1, 8 cycles as index read 1, and 8 cycles as index read 2) according to the manufacturer’s instructions (Illumina Inc.). GBS libraries were sequenced at the facilities of the Leibniz Institute of Plant Genetics and Crop Plant Research (IPK) in Germany, aiming for a minimum coverage of at least 40×. Compared to the protocol used by Shekhovtsov et al. [28], our GBS libraries differed in two main aspects. While both studies applied the same restriction enzymes (*Pst*I and *Msp*I), Shekhovtsov et al. used a broader size selection range (240–600 bp) and sequenced the libraries on an Illumina NextSeq 500 platform. In contrast, our libraries were size-selected for 400–600 bp fragments and sequenced using the Illumina NovaSeq 6000. These differences were taken into account during data processing and filtering prior to merging the datasets.

### GBS data preparation and filtering

Sequences were de-multiplexed using the Casava pipeline 1.8 (Illumina Inc.) and the restriction site and barcode were trimmed from all GBS sequence reads using Cutadapt [45] within ipyrad v.0.9.58 [46]. The initial quality assessment of all raw sequence samples was performed using FastQC [47] to identify overrepresented reads, remaining adapter sequences, and to determine appropriate trimming thresholds. Reads shorter than 65 bp after adapter removal were discarded. Eleven samples of *Tilia* GBS data of Shekhovtsov et al. [28] (NCBI BioProject: PRJNA811982) were analyzed together with our GBS samples. Clustering of sequence data was performed using the ipyrad bioinformatics pipeline, with a minimum coverage threshold of 6× and a clustering threshold 0.90. Default settings were used for all other parameters. The GBS data assembly was performed *de novo*. The complete data set was split into a “species” dataset for phylogenetic analysis and a dataset comprising Iranian samples, *T. dasystyla*, its hybrid taxon *T. × euchlora*, plus *T. platyphyllos* as outgroup for population genetic analysis of Hyrcanian *Tilia* individuals. SNPs were checked for their patterns by examining allele frequency histograms and verifying the absence of multi-allelic loci or artifacts, and were found to be nearly completely bi-allelic, even in tetraploid taxa. Consequently, SNP calling was conducted in ipyrad, which is configured for diploid taxa, i.e. bi-allelic SNPs. We then utilized VCFtools v 0.1.16 [48] for additional filtering for both data sets removing all indels, keeping only sites with minor allele frequency of 0.02, allowing up to 30% missing data at a site, a minimum depth of 10 and a maximum depth of 300.

### Determination of genomic heterozygosity

While the majority of SNPs in polyploids are typically bi-allelic, the degree of heterozygosity increases with ploidy level in outcrossing autopolyploid species and alloploids [49, 50]. Therefore, it can be used as a proxy for ploidy levels. Heterozygosity of the individuals was determined using DnaSP v6 [51], with vcf files generated with ipyrad as input. The DnaSP output was used to calculate the ratio of heterozygous sites to the total number of sites of the samples and was correlated with the measured genome sizes to identify ploidy levels.

### Phylogenetic analyses

For initial tests of the dataset, we conducted neighbor-joining (NJ) analyses in Paup* 4.0a169 [52] using the general time-reversible model of sequence evolution (GTR) with rates following a gamma distribution with a shape parameter of 0.5 to calculate pairwise genetic distances.

We used two datasets in the final phylogenetic analyses. One consisting of all species but including just seven Iranian individuals selected to represent different geographic areas and the main genetic clusters inferred from initial population structure analyses to represent the genetically rather narrow Iranian *Tilia* populations. In this analysis we defined *T. americana* L. as outgroup, as it is clearly outside of the group of Eurasian taxa we analyzed and because we had no access to *T. endochrysea* Hand.-Mazz. that is sister to all other species within the genus [12]. The second dataset was used to explore population structure within Hyrcanian *Tilia*. For this dataset we included all 85 individuals from Iran together with their closest relatives according to the analysis before, i.e. a cultivated individual of *T. dasystyla* obtained from the Botanical Garden Munich, *T. × euchlora* as an assumed hybrid involving *T. dasystyla* as one parent, and *T. platyphyllos.* The latter species was defined as outgroup. For this second dataset we tested also network approaches in SplitsTree App [53] and IQ-Tree v2.2.6 [54, 55] including *T. dasystyla* from the Botanical Garden Munich as outgroup to account for potential reticulate relationships.

Both datasets were analyzed using IQ-Tree to calculate species relationships in a maximum-likelihood (ML) framework and Paup* for parsimony analyses (MP). As input file for IQ-Tree the filtered vcf file obtained by VCFtools was converted into Phylip format using the Python script vcf2phylip.py [56]. Modelfinder [57] was employed to find the best-fit model for phylogenetic inference, determined by the Bayesian information criterion for the ML analysis, resulting in the transversion model with equal base frequencies (TVME). We used 1000 bootstrap replications [58] to infer clade support with a burn-in of 250.

For MP analyses of both GBS datasets we used two-step heuristic searches, as described in Blattner [27], with tree-bisection-reconnection (TBR) branch swapping, steepest descent not in effect, and initial 5000 random addition sequences (RAS) restricting the search to 50 trees per replicate, keeping only shortest trees. The resulting trees were afterwards used as starting trees in a search with maxtree set to 10,000 if the limit of 50 trees was hit during a replicate in step one. To test clade support, 1000 bootstrap re-samplings were conducted with the same settings as before, except that we did not use the initial RAS step.

### Population structure analyses

For *Tilia* from the Hyrcanian Forest we used population assignment analysis in the R package ‘LEA’ [59] employing sparse non-negative matrix factorization (sNMF) together with principal component analysis (PCA) to identify sub-structuring of the populations. Population assignment was performed for K = 1–10 with 30 repetitions each. The cross-entropy plot (Supplementary Fig. S1) was examined to infer the K with the lowest value and validate the robustness of the selected K. The Q-matrices from ‘LEA’ were sorted using the R package ‘tidyverse’ [60] according to longitude and visualized with ‘ggplot2’ [61], discerning different ancestral clusters by color-coding. PCA results obtained from ‘LEA’ were also visualized with ‘ggplot2’ in R.

Additionally, we inferred coancestry patterns by examining how many alleles are shared between individuals at loci derived from GBS data. Here, we used Stacks v2.55 [62] to generate a RADpainter input file for fineRADstructure v0.3.2r109 [63] for the Hyrcanian limes. In Stacks, a locus needed to be present in 80% of the individuals of a population and in 50% of all individuals to be processed. The initial vcf file was filtered using VCFtools as described above and taken as input for Stacks*population* to generate a RADpainter format. Shared coancestry patterns were inferred using 10,000 burn-in steps in the Monte Carlo Markov Chains (MCMC) analysis with 100,000 further iterations with keeping every 1000th sample. This run was continued, adding additional 100,000 steps, treating the original run as burn-in. To get the best posterior state for the tree, 1000 attempts were used. Results were visualized in R with the functions provided by fineRADstructure.

## Results

### Analysis of genome sizes and determination of ploidy levels

Flow cytometry was used to measure genome sizes and infer ploidy levels of the species in this study (Table S1). For diploid species we measured 2C values of about 2 pg DNA, while tetraploid genomes resulted in 2C values between 4 and 5 pg DNA. We found that *T. dasystyla*, the measured samples from Iran, and *T.* × *euchlora* were tetraploid, which confirms earlier reports of 2*n* = 4*x* = 164 chromosomes for *T. dasystyla* and *T.* × *euchlora* [10]. All other measured species in this analysis turned out to be diploid. As we did not measure all Iranian individuals by flow cytometry, we used genomic heterozygosity as a proxy for polyploidy and compared it to the respective values of its closest diploid relative *T. platyphyllos* (Table S2). For the diploid taxon we obtained values between 0.0339 and 0.0397 with an average of 0.0370. For the Iranian individuals the values were in a range from 0.0542 to 0.0681 with an average of 0.0623, while *T. dasystyla* from Munich Botanical Garden had a heterozygous fraction of 0.0674 and *T.* × *euchlora* of 0.0690. From this we deduce that not only the individuals measured by flow cytometry but all Iranian *Tilia* individuals in our study are tetraploid.

### Genotyping-by-sequencing

In total, our GBS de-novo assembly produced 32,973 ± 12,552 clusters per sample with a depth of 6× or higher (for the Shekhovtsov et al. [28] samples included here we obtained 230,446 ± 68,192 clusters). The data set for the Eurasian *Tilia* phylogeny comprised 37 samples and consisted of 9169 loci with 20.40% missing data. The alignment of concatenated loci had a length of 1,038,386 bp with 96.7% constant characters. Out of the 35,548 variable characters, 23,011 were parsimony-informative. The SNP matrix that served as an input for IQ-Tree contained 25,044 SPNs of which 12,014 were parsimony-informative, 5922 singleton sites and 7108 constant sites. The SNP matrix for population analysis comprised 85 *T. dasystyla* samples and 13,404 SNPs after filtering with VCFtools. The number of raw sequence reads per individual ranged from ~ 2 to 23 million for the Shekhovtsov et al. samples, and from 1 to 3 million in our dataset. These values were comparable and were accounted for during filtering and downstream analyses.

### Phylogenetic inference

In an initial NJ analysis, we included *T. americana*, *T. amurensis*, *T. begoniifolia*, *T. cordata*, *T. dasystyla*, *T. × euchlora*, *T. × europaea* L., *T. platyphyllos*, *T. tomentosa* Moench., *T. taquetii*, and all Iranian samples. The analysis clearly separated Iranian *Tilia* as a monophyletic group from other individuals and showed that they internally are all very similar in comparison to the variation within other species (Fig. S2). The average pairwise genetic distance between Iranian individuals was around 0.14% while for, e.g., *T. platyphyllos* individuals, distances were between 0.12 and 0.28% and for *T. tomentosa* 0.09 to 0.23%. For an analysis of species relationships, we therefore excluded most of the Iranian individuals using just seven of them to represent the taxon.

**Fig. 1:**
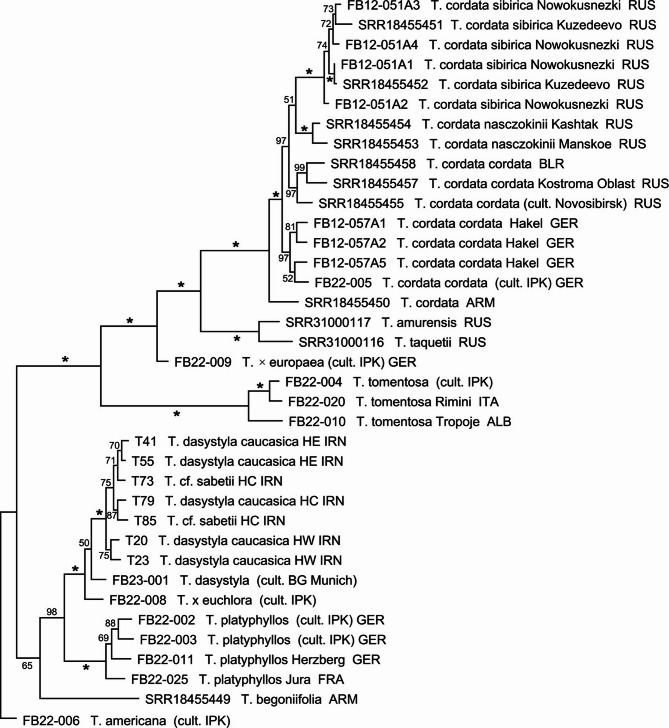
Phylogeny of Eurasian *Tilia *taxa**.** The ML tree is based on the GBS dataset including all taxa. Numbers along branches provide bootstrap values (%) with asterisks depicting 100% support. *Tilia americana* was defined as outgroup. Data for accession numbers starting with “SRR” were taken from Shekhovtsov et al. [28]

Analyses of the “species” dataset by ML and MP resulted in very similar tree topologies (Figs. 1 and S3) with high support values along the backbone of the trees. We found essentially three groups within Eurasian *Tilia*. One consisting of *T. begoniifolia* as sister to *T. platyphyllos*, *T. × euchlora*, and *T. dasystyla*, the latter including the seven analyzed Iranian individuals. A second clade consisted of southern European *T. tomentosa*. At the base of the third group is the widely cultivated *T. × europaea*, an assumed homoploid hybrid between *T. cordata* and *T. platyphyllos* that is regularly planted in settlements and along alleys in Central Europe. The next of the consecutively branching clades consists of *T. amurensis* together with *T. tarquetii* from Russian Far East, followed by the large *T. cordata* clade divided in several subspecies. Interestingly, in this latter group our own collection of subsp. *sibirica* samples forms a maximally supported clade with the individuals of the same taxon that were analyzed by Shekhovtsov et al. [28], clearly indicating that different GBS datasets can be combined for extended phylogenetic analyses (Fig. 1).

From all species reported to occur in Iran, based on our sample we could only confirm *T. dasystyla* to be present. If the before reported other species would occur in Iran, they should group in phylogenetic trees with their conspecific individuals, which is the case for *T. dasystyla* but for none of the other taxa. Moreover, the Iranian individuals form a genetically tight group that makes it very unlikely that multiple species are involved when compared to genetic diversity in other species where multiple individuals were analyzed (see above).

**Fig. 2:**
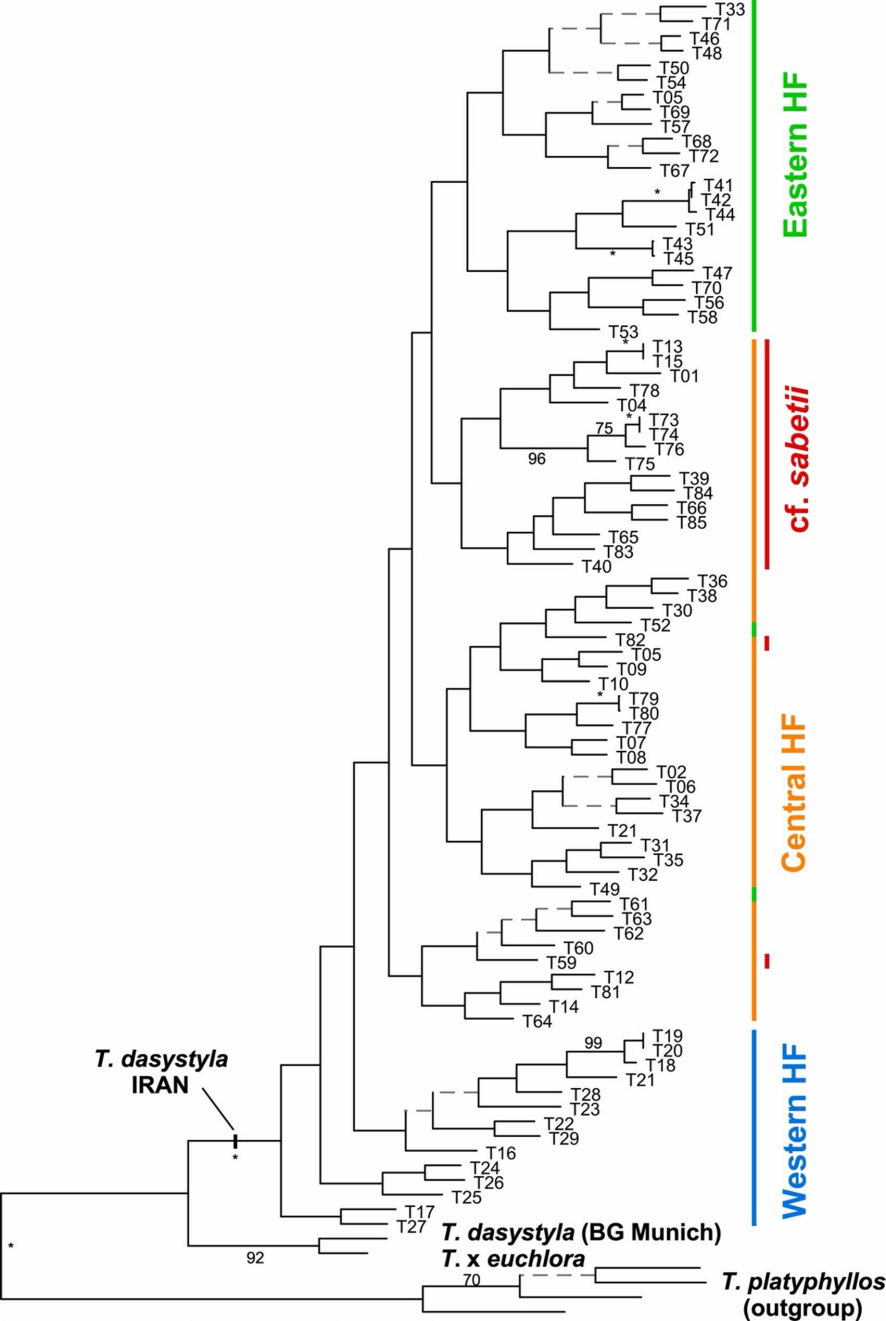
Phylogeny of Hyrcanian *Tilia***. ** One of 48 equally parsimonious trees of an MP analysis of GBS data for the Iranian *Tilia *individuals plus closest relatives. *Tilia platyphyllos* was defined as outgroup. Clades collapsing in the strict consensus tree are indicated by dashed gray lines. Numbers along branches provide bootstrap values (%) with asterisks depicting 100% support. HF stands for Hyrcanian Forest

**Fig. 3:**
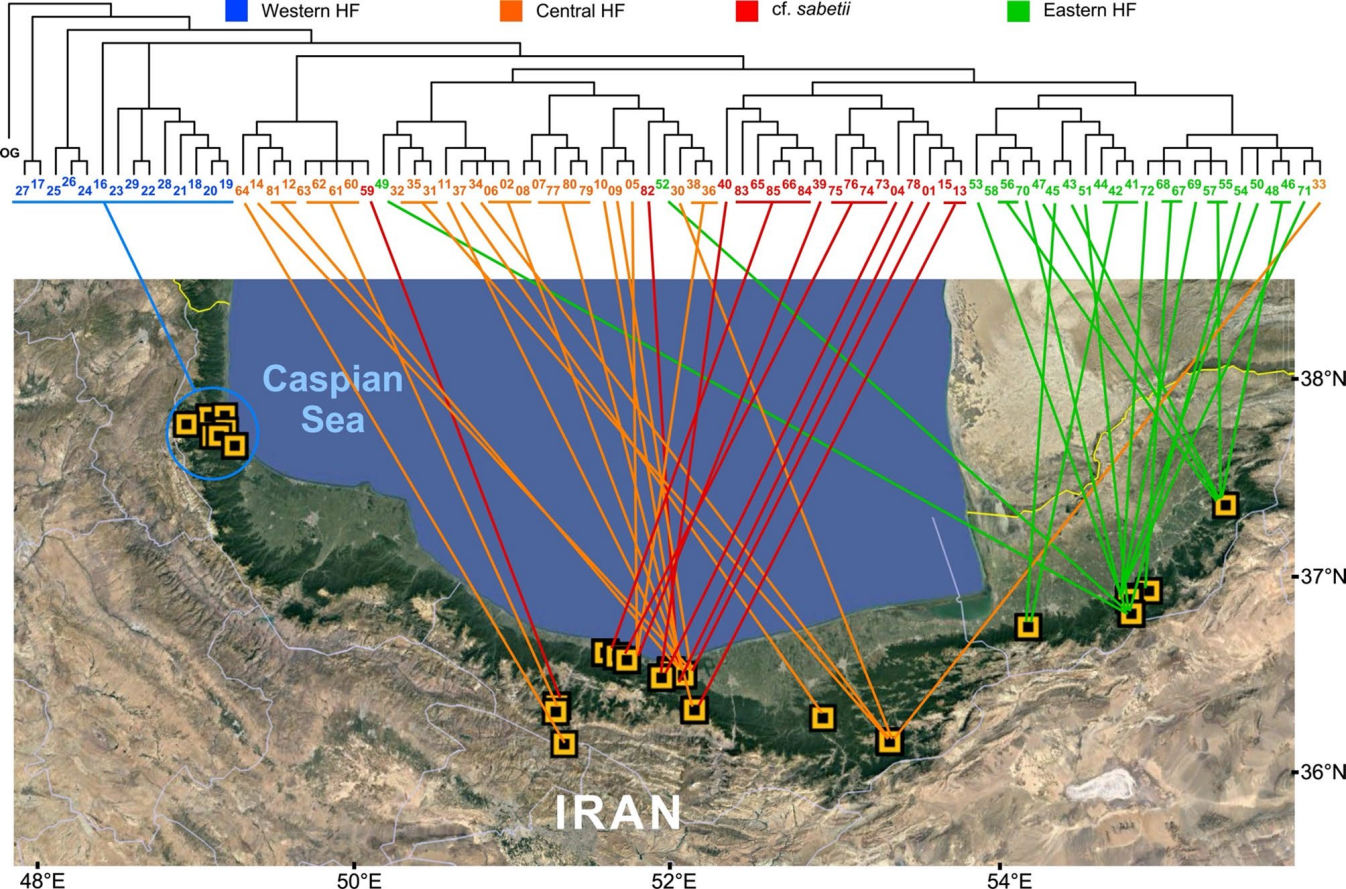
Phylogeography of Hyrcanian *Tilia*. The plot correlates the phylogenetic positions of individuals in the strict consensus tree of the MP analysis with their collection sites in the western, central and eastern parts of the Hyrcanian Forest (HF). The satellite map of the area of the southern Caspian Sea was taken from Google Earth

In additional analyses we used the dataset consisting of all Iranian individuals plus their closest relatives, i.e. cultivated *T. dasystyla*, *T. × euchlora*, and *T. platyphyllos* to analyze population structure of *Tilia* within the Hyrcanian Forest. This dataset had 7931 variable sites of which 4241 were parsimony informative. *Tilia platyphyllos* was defined as outgroup for parsimony analysis, resulting in 48 equally parsimonious trees of 19,301 steps length (consistency index, CI = 0.41; retention index, RI = 0.59). In this analysis *T. × euchlora* together with the individual cultivated in Munich Botanical Garden of *T. dasystyla* are sistergroup to all Iranian individuals of *T. dasystyla* (Fig. 2). Within the latter we see a clear geographic pattern with progression of clades from west to east (Figs. 2 and 3). The western populations form a basal grade in the phylogenetic tree, harboring the central and eastern populations. The eastern populations (marked green) form a clade within the central populations, except for the individuals T49 and T52, which were collected in Golestan but belong to the central Hyrcanian genotypes. Individual T33 clearly is an eastern genomic type that was collected on the easternmost reaches of the otherwise central Hyrcanian populations (Fig. 3). These cases indicate that some gene flow between the central and eastern populations occur. There is no evidence or historical record suggesting human-mediated movement of individuals between these regions. Given the forest structure and inaccessibility of many sampling sites, we consider natural dispersal the more likely explanation for the observed gene flow. The western populations, although being the initial source populations for the more easterly stands, are today genetically and geographically clearly separated. Low amounts of gene flow and hybridization is also evident from the highly resolved strict consensus tree (Fig. 3) where polytomies, which in MP often are indicative of mixed ancestry, are restricted to mostly the terminal nodes within the geographic groups while the backbone of the tree is well resolved.

Sixteen out of 18 individuals characterized by their spoon-shaped bract, form a clade in the MP analysis (marked in red) within the populations from the central Hyrcanian Forest that is sister to all populations from the east (green), while in ML they are polyphyletic within the central Hyrcanian group (Fig. S5). To these individuals we refer here as cf. *sabetii*, as they resemble in their morphology mainly *T. sabetii* of Zare et al. [8]. Individuals T59 and T82 have the cf. *sabetii-*like bracts but fall in the genomic group of central Hyrcanian subsp. *caucasica* individuals in MP (Fig. 3). Even if this might indicate gene flow between both types, hybridization seems generally rare according to the GBS data, as both types grow often in close proximity. To see if other assumed Iranian *Tilia* taxa form genomically monophyletic groups, we plotted our initial species determinations on the phylogenetic tree shown in Fig. 2. This was, however, not the case (Fig. S4). All assumed species form polyphyletic groups, with individuals widely dispersed over the tree

To account for potential reticulate structures among the populations in this dataset we analyzed the sequences with neighbor net in SplitsTree App and build consensus networks based on ML bootstrap trees obtained from IQ-Tree. Both approaches identified low amounts of reticulations, inferring an overall tree-like structure of relationships among individuals. Exemplarily a consensus network is provided in Figure S5 derived from 750 ML trees, indicating four network parts with small reticulations. The main difference towards the MP analysis regards the cf. *sabetii* individuals, which are distributed in multiple polyphyletic clusters throughout the central Hyrcanian populations instead of forming mainly one cluster as in MP.

### Population genetic analyses

**Fig. 4:**
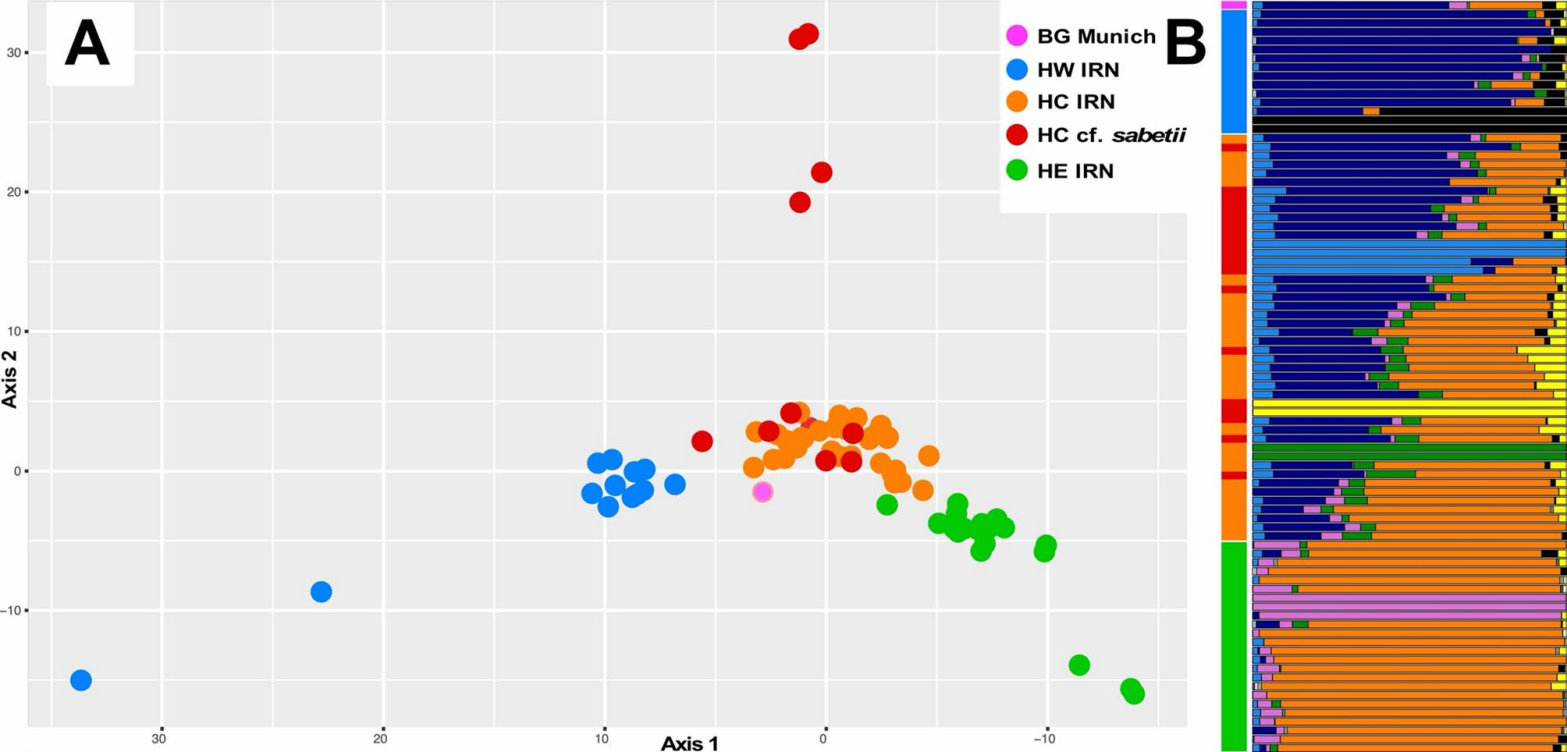
Population analyses of Hyrcanian *Tilia***. A **PCA plot of the first two principal components, explaining 56.1% of the genetic variation, of the GBS-based data of Hyrcanian *T. dasystyla* populations. **B** Population assignment analysis for the same dataset at K = 7 ordered according to the longitudinal occurrence of the individuals from west (top) to east (bottom). The vertical color bar to the left provides the individuals’ group affiliations according to the legend for panel (**A**), while the horizontal bars in seven colors within the LEA plot show the proportion of ancestry coefficients at K = 7 for each individual

Similar to the phylogenetic analyses, also population genetic methods reflect the geographic patterning of the Hyrcanian *T. dasystyla* populations. In the PCA (Fig. 4A) the west to east aligned geographic groups separate along PCA Axis 1, explaining 30.4% of the genetic variation. The cf. *sabetii* individuals group with the central Hyrcanian individuals but partly spread out in addition along Axis 2, which explains 25.7% of the genetic variation.

Bayesian population assignment analyses indicates that hierarchical structuring of the Hyrcanian populations through increasing K values between two and seven (Fig. S6) is generally weak. The Evanno method indicated K = 7 as best representation of the population structure. The respective plot is shown in Fig. 4B, where a gradient from west, dark blue in the upper part of the figure, to east, orange in the lower part, can be recognized. The outlier/extreme individuals in the PCA are here characterized by nearly completely different color bars: black in the western group, blue and yellow in cf. *sabetii*, and pink in the eastern population. The two individuals with green bars in the population assignment plot belonging to the central Hyrcanian population are not obvious in the PCA. The cf. *sabetii* individuals as a group have no distinguishing pattern of their own in this analysis. This fact is also reflected in the fineRADstructure analysis of the GBS data where the simple coancestry matrix did not result in clear differentiation of cf. *sabetii* from other individuals within subsp. *caucasica* (Fig. S7). Generally, the Iranian *T. dasystyla* populations were all appearing to be approximately equidistant (shared coancestry values varied between 89 and 139, with a mean of 92).

## Discussion

### Merging GBS datasets

The advantage of using sequences of genes or specific marker regions in phylogenetics and population genetics is their additivity, i.e. each new sequence can be added to the already existing stock of earlier sequences stored in nucleotide databases. Thus, they can be analyzed together, no matter which lab produced the sequences initially [64]. In contrast, anonymous fragment-based markers like, e.g., amplified fragment lengths polymorphisms (AFLP) or microsatellites (SSR), are not additive. This means results from different labs can normally not be easily combined into single datasets. GBS data are somehow in between. Although the fragments are clearly defined through their restriction enzyme cutting sites, their positions in the genome are unknown if not mapped to a reference genome of the analyzed species. However, as GBS fragments are sequenced, their sequences are always known, and normally clustered during the analysis according to sequence homology. We took advantage of this information and combined our own GBS data with a dataset [28] produced using identical restriction enzymes (*Msp*I, *Pst*I) but with slight differences in fragment size selection (240–600 bp vs. 400–600 bp) and sequencing platforms (Illumina NextSeq 500/550 vs. Illumina NovaSeq 6000). As no nuclear genome of the analyzed *Tilia* species is available, both studies used *de novo* approaches, i.e. analyzing the data without initial mapping of GBS reads to a reference genome. We downloaded the Shekhovtsov et al. raw reads from GenBank and processed them together with our own reads in the ipyrad pipeline. The fraction of reads derived from potentially shorter fragments in this dataset did play no role in the analyses, as they were present in only a small number of individuals and therefore removed during filtering. The phylogenetic analyses showed, that *T. cordata* subsp. *sibirica* members, which were included in both datasets, form a strongly supported clade in the phylogenetic trees, with no obvious bias that would result in clustering the individuals used by Shekhovtsov et al. and our own materials in separate subclades within subsp. *sibirica* (Fig. 1). As it is, this is the result we expected if the datasets would be compatible, as subsp. *sibirica* populations are small, marginal populations separated for some time from their sources in the west [28], and are genetically not very diverse.

In addition to being additive, the GBS results clearly indicate that within this genetically not very diverse genus, the method is able to separate species and subspecies and detect even geographic patterns in populations. This will allow scientists to use additional GBS studies to infer the relationships within other geographic subgroups, like the American or East Asian *Tilia* species, and afterwards combine different datasets to arrive at a comprehensive phylogeny, at least for the diploid species and their subspecific taxa in the genus. To include allopolyploids, initial phasing would be necessary [12] that is currently not easily done with GBS data when reference genomes are absent. Moreover, merging independent GBS datasets seems a promising community approach also for other widely distributed taxa, given that the same restriction enzymes are used and raw sequence data made available.

### Tilia phylogeny

We essentially follow here the monograph of Pigott [10] regarding *Tilia* species numbers and taxon names, as he took the entire genus into account in contrast to the restricted local approaches published in diverse floras. However, it is obvious that without a unified molecular account of the genus it will not be possible to finally clarify the taxonomy of *Tilia*. Thus, an individual determined as *T. begoniifolia* from Armenia included in the Shekhovtsov et al. [28] dataset, for sure does not belong to *T. dasystyla* subsp. *caucasica* (Fig. 1), where Pigott [10] lists *T. begoniifolia* as a synonym, but is sister to the *T. platyphyllos* group. For this taxon, the determination of ploidy level would be of interest. On the other hand, Hyrcanian individuals determined by us as *T. begoniifolia* could not be differentiated from *T. dasystyla* subsp. *caucasica* in our GBS analysis, which supports Pigott’s view. Based on our experience here, one has to include multiple individuals representing multiple populations of a *Tilia* taxon to be able to find species borders and arrive at a meaningful phylogeny, as most morphological characters are obviously too variable to define taxa in an evolutionary context in this genus.

The results of our phylogenetic analyses confirm the species relationships provided byXie et al. [12] for the species we included. Thus, *T. platyphyllos* is separated from the *T. cordata* and the *T. tomentosa* groups. Within the *T. cordata* group, *T. amurensis* and *T. taquetii* together are sister of *T. cordata*. Both species are close relatives so that Pigott’s treatment of *T. taquetii* as a subspecies of *T. amurensis* seems possible ([10, 25]. Diploid *T. platyphyllos* is a close relative of tetraploid *T. dasystyla* in our phylogenetic tree, which supports the results of Xie et al. [12] who identified *T. platyphyllos* and a species close to *T. cordata* as possible parents of the tetraploid. Also *T. × euchlora* groups with *T. dasystyla*, confirming the possibly similar parental descent of these allopolyploids [10]. *Tilia tomentosa* individuals form a group of their own in our analysis, which is also consistent with Xie et al. [12], where this southern European species has its closest relatives among Chinese *Tilia* taxa, which were not included here.

On the level of subspecies, the addition of our *T. cordata* individuals from Central Europe to the dataset provided by Shekhovtsov et al. [28] did not change their results and conclusions regarding the subspecies of this taxon. Thus, the Russian subspp. *sibirica* and *nasczokinii* are part of the *T. cordata* clade, and their *T. cordata* individual from Armenia is sister to all other *T. cordata* individuals, and possibly groups outside of subsp. *cordata*. The conclusion of Shekhovtsov et al. [28] that this could be another taxon within *T. cordata* therefore remains. For *T. dasystyla*, we have had no access to individuals from *T. dasystyla* subsp. *dasystyla* occurring in Crimea, and subsp. *multiflora* (Ledeb.) Pigott from the Caucasus foothills close to the Black Sea. Here additional population studies seem desirable to see how different these subspecies are in comparison to the ones defined within *T. cordata*. This would also help to further improve understanding of the status of Hyrcanian limes.

### Hyrcanian Tilia taxa

For the Hyrcanian Forest between one and seven *Tilia* species have been proposed to occur. We here used for the first time a phylogenomic approach together with thorough sampling of most populations present in this area to test these assumptions. We found the individuals in our analysis occurring in the Hyrcanian Forest to be genetically very closely related (Figs. S2, S7) and all falling in one clade that corresponds to *T. dasystyla* subsp. *caucasica* (Fig. 2). This essentially confirms the view of Pigott [10] who determined only this taxon to be present in Iran. As we included all other *Tilia* species from western Eurasia in our analysis and none of the Iranian samples did group with any of these species, we can clearly state that except of *T. dasystyla* none of these species is present in Iran. However, within our Hyrcanian Forest population samples, we included a group of individuals that resemble the narrow Hyrcanian endemic *T. sabetii* of Zare et al. [8]. *Tilia sabetii* is characterized by the spoon-like shape of the free, upper parts of its bracts, which are conspicuously different from the mainly parallel or narrowing bracts of other *Tilia* species. To these trees we here refer as cf. *sabetii*. They occur in sympatry with *T. dasystyla* subsp. *caucasica* in parts of the central Hyrcanian Forest (Fig. 5) and are in our population genetic analyses not differentiated from this taxon (Figs. 6, S5, S7). For *T.* cf. *stellato-pilosa*, another Iranian endemic taxon, we can currently arrive at no safe conclusion, as only three individuals within our collection were initially determined as belonging to this taxon. However, as the cf.*stellato-pilosa* individuals are widely dispersed over the phylogenetic tree (T33, T34 and T35 in Fig. 2), the status as an evolutionary unit is highly questionable. Neither of the species assumed to occur in Iran are monophyletic in the phylogenetic analysis (Fig. S5) according to our determinations, which led us to treat them as ecotypes of *T. dasystyla* subsp. *caucasica*, and exclude them from further taxonomic discussion here.

This is different for the cf. *sabetii* individuals. Here a morphologically distinct type was before described as a separate species [8] and forms with only two exceptions (T59, T82) a clade within *T. dasystyla* subsp. *caucasica* in the MP analysis. This would qualify this taxon to be assigned a new rank and recognized as *T. dasystyla* subsp. *sabetii*. However, as cf. *sabetii* and subsp. *caucasica* occur in mixed stands, this treatment would violate the requirement of mainly separate distribution areas for subspecies: Belonging to the same species would imply that fertile offspring should be possible, which would readily blur the differences discerning both taxa if growing in sympatry. Thus, if these taxa are reproductively isolated, they would fulfill the prerequisite of the biological species concept [65, 66], asking to recognize them as separate species. In this case, *T. sabetii* would in the phylogenetic tree be part of subsp. *caucasica*, rendering the latter taxon paraphyletic. While paraphyly should generally be avoided in systematics, it is a natural process in evolution [66, 67], where paraphyletic groups might originate and persist for some time within other taxa. Through time and with ongoing diversification and extinction, paraphyly will in most cases resolve into monophyly. However, this is not always the case and some paraphyletic groups might persist for long evolutionary timespans [68]. With regard to the cf. *sabetii* individuals we have currently not enough knowledge to decide their taxonomic status, particularly as they are, in contrast to MP, in ML and network analyses dispersed within the central Hyrcanian cluster. A first step would be a careful analysis of mixed stands to identify potential F1-hybrid individuals to infer the mode of inheritance of the distinguishing bract character.

Within Hyrcanian *Tilia* we found a basic grade in the phylogenetic trees consisting of populations from the west, followed by a central Hyrcanian grade that includes cf. *sabetii* individuals and a clade formed by populations from the eastern reaches of the Alborz Mts. in Golestan (Fig. 5, S4). These relationships were also detected in ML analyses and clearly reflect a west to east colonization throughout the Hyrcanian Forest, pointing to the Caucasus foothills as potential source of *T. dasystyla*. This is in accord with the highest taxonomic diversity occurring in this region, where the three subspecies recognized within *T. dasystyla* meet. However, here we don’t have molecular data to substantiate this assumption. A west to east colonization pattern in the Alborz region was detected earlier [33] in Persian poppy (*Papaver bracteatum* Lindl.). If this reflects a certain ecoclimatic setting in the present [7] or past that made the western parts of the Hyrcanian Forest earlier habitable for *Tilia* than the eastern Golestan region we can currently not infer, as this would need more comparative studies in a wider range of taxa from this area. As genetic differentiation among Hyrcanian *Tilia* populations is low, this may reflect on the one hand slow differentiation in long-living organisms like lime trees, but can also be attributed to ongoing gene flow facilitated by the relatively continuous forest cover and the absence of major geographic barriers across the region. Human-mediated gene flow seems unlikely here, given the distribution and remote habitats of the *Tilia* populations and the absence of systematic forestry in these parts of northern Iran. Generally, the Hyrcanian landscape shows considerable ecological heterogeneity along altitudinal and longitudinal gradients, including variation in precipitation, temperature, soil types, and forest composition [3, 38]. It remains possible that microhabitat differences contribute to subtle ecological differentiation, particularly in morphologically divergent but genetically similar individuals such as cf. *sabetii*. The sympatric occurrence of these taxa suggests that ecological factors can play a role in maintaining phenotypic variation despite limited genomic divergence. Furthermore, the west-to-east pattern observed in the phylogenetic trees may partly reflect ecological gradients, as the western Hyrcanian Forest is wetter and more topographically complex than the eastern region, potentially offering earlier or more suitable conditions for colonization ([33, 39]. These hypotheses remain to be tested with targeted ecological-genetic integration in future studies.

## Conclusions

Our data clearly show the advantage of using multiple individuals and populations in molecular studies to find possible species borders within *Tilia*, which might be the only way to finally overcome the taxonomic confusion connected to the taxa in this genus. The additivity of GBS datasets will allow merging individual studies of populations of *Tilia* subgroups from labs in different geographic areas into a larger analysis to finally arrive at a complete taxon representation. Thus, we propose to use the same GBS protocol provided here in future phylogeographic studies of *Tilia*. More generally, merging data from multicentric studies might be a way to overcome the rising bureaucratic hurdles, complicating material exchange even for taxonomic studies.

Regarding the Iranian *Tilia* individuals included in our analysis, we show that they all fall within a narrow clade consistent with *T. dasystyla* subsp. *caucasica* and that none of the other Eurasian lime species occurs in this area. For *Tilia*, the Hyrcanian Forest does therefore not qualify as Pleistocene ice-age refugium from where higher latitudes of Eurasia were re-colonized after climate warming. Individuals morphologically conforming *T. sabetii*, a species described to be endemic for the central Hyrcanian Forest, form a polyphyletic group within subsp. *caucasica*. Both types occur in sympatry so that their taxonomic status is currently undecided. Treating cf. *sabetii* individuals as a species would make *T. dasystyla* paraphyletic, while sympatric subspecies should not occur, as gene flow would soon obliterate the differences between them. Here further tests for reproductive isolation seem interesting.

## Supplementary Information

Additional File 1: Fig. S1. Cross-entropy evaluation to arrive at an optimal number for K in population assignment analysis. Cross entropy values are plotted for K = 3 to 10 with a lowest value at K = 7. Fig. S2. Neighbor-joining tree for initial inference of genetic distances for the individuals in the GBS analyses of *Tilia*. The tree indicates that all Iranian individuals in the analysis form a genetically narrow and monophyletic group. Fig. S3. Phylogenetic analysis of the GBS-derived “species” dataset by MP resulted in two equally parsimonious trees. Branches collapsing in the strict consensus tree are provided as dashed gray lines. Numbers along branches are bootstrap values (%) derived from 1000 bootstrap re-samples, with asterisks indicating 100% support. The tree length is 52,780 steps (CI = 0.68, RI = 0.86). Fig. S4. Initial species determinations (abbreviated after individual numbers and color coded) for the Hyrcanian *Tilia* individuals plotted on the parsimony tree of Figure 1 showing that none of the taxa form a monophyletic group. Fig. S5. Consensus network based on 750 ML trees of Hyrcanian *Tilia dasystyla* populations. An individual from the Botanical Garden Munich was included as outgroup. Colors code the geographic regions with western populations (blue), central Hyrcanian populations (orange), and eastern populations (green). Red color indicates cf. *sabetii* individuals occurring within the central Hyrcanian Forest stands. Arrows depict the four parts where reticulate relationships were inferred, although overall reticulation is low and relationships are mostly tree-like. Numbers of individuals (Supplementary Table S1) are provided for reticulate structures in mixed clusters of *cf. sabetii* and central Hyrcanian subsp. *caucasica* individuals. Fig. S6. Bayesian population assignment analyses at K= 2 to K = 7. The individuals are ordered longitudinal, with the individuals from the west on the left and the eastern individuals to the right. Hierarchical substructuring is weak in the data and the west to east pattern of colonization becomes only clear at K = 6 and K = 7. Fig. S7. Simple coancestry matrix derived from an analysis of the GBS data of the Iranian *Tilia* individuals visualized in fineRADstructure. Individuals were ordered longitudinally. The plot shows that differentiation between individuals in Iran is generally low and that cf. *sabetii* individuals do not form a genetic group of their own. Colors of labels correspond to sampling regions: blue = western Hyrcanian (HW IRN), orange/red = central Hyrcanian (HC IRN), and green = eastern Hyrcanian (HE IRN), while red indicates individuals determined as cf. *sabetii*. Table S1. *Tilia *materials analyzed in this study. Herbaria are abbreviated according to Index Herbariorum as GAT (Institute of Plant Genetics and Crop Plant Research, IPK Gatersleben, Germany), OSBU (Herbarium of the Botanical Institute of the University of Osnabrück, Germany), M (Botanische Staatssamlung und Botanischer Garten München, Munich, Germany) and HUI (Herbarium of the University of Isfahan, Iran). Table S2. Analysis of heterozygosity to discern di- and tetraploidy in the set of Iranian individuals.

## Data Availability

The dataset supporting the conclusions of this article is available in the European Nucleotide Archive (ENA) under project PRJEB80134. The aligned concatenated GBS sequences used in the phylogenetic analyses [69] can be retrieved through e!DAL [70] via 10.5447/ipk/2025/10. Herbarium vouchers of the analyzed materials were stored in public herbaria according to Supplementary Table S1.

## Abbreviations

bp: Base pairs
GBS: Genotyping-by-sequencing
GTR: General time-reversible model
HF: Hyrcanian Forest
IPNI: International Plant Names Index
ML: Maximum-likelihood analysis
MP: Maximum-parsimony analysis
NJ: Neighbor-joining analysis
PCA: Principal-component analysis
pg: Picogram
PI: Propidium iodide
RAS: Random addition sequences
SNP: Single-nucleotide polymorphism
TBR: Tree-bisection-reconnection
