# Supplementary material for: Phylogenomics of Western Eurasian Tilia: merging GBS datasets to place the Hyrcanian forest limes
Source: BMC Plant Biol. 2025 Oct 14;25:1370. doi: 10.1186/s12870-025-07435-4 (PMC12522541; doi:10.1186/s12870-025-07435-4)
Supplement: Supplementary file 1 — Additional File 1: Fig. S1. Cross-entropy evaluation to arrive at an optimal number for K in population assignment analysis. Cross entropy values are plotted for K = 3 to 10 with a lowest value at K = 7. Fig. S2. Neighbor-joining tree for initial inference of genetic distances for the individuals in the GBS analyses of Tilia. The tree indicates that all Iranian individuals in the analysis form a genetically narrow and monophyletic group. Fig. S3. Phylogenetic analysis of the GBS-derived “species” dataset by MP resulted in two equally parsimonious trees. Branches collapsing in the strict consensus tree are provided as dashed gray lines. Numbers along branches are bootstrap values (%) derived from 1000 bootstrap re-samples, with asterisks indicating 100% support. The tree length is 52,780 steps (CI = 0.68, RI = 0.86). Fig. S4. Initial species determinations (abbreviated after individual numbers and color coded) for the Hyrcanian Tilia individuals plotted on the parsimony tree of Figure 1 showing that none of the taxa form a monophyletic group. Fig. S5. Consensus network based on 750 ML trees of Hyrcanian Tilia dasystyla populations. An individual from the Botanical Garden Munich was included as outgroup. Colors code the geographic regions with western populations (blue), central Hyrcanian populations (orange), and eastern populations (green). Red color indicates cf. sabetii individuals occurring within the central Hyrcanian Forest stands. Arrows depict the four parts where reticulate relationships were inferred, although overall reticulation is low and relationships are mostly tree-like. Numbers of individuals (Supplementary Table S1) are provided for reticulate structures in mixed clusters of cf. sabetii and central Hyrcanian subsp. caucasica individuals. Fig. S6. Bayesian population assignment analyses at K= 2 to K = 7. The individuals are ordered longitudinal, with the individuals from the west on the left and the eastern individuals to the right. Hierarchical subs [file 12870_2025_7435_MOESM1_ESM.pdf]

Phylogenomics of Western Eurasian *Tilia*: Merging GBS datasets to place the Hyrcanian Forest limes

Nastaran Ala, Ali Bagheri, Habib Zare, Axel Himmelbach, Dörte Harpke and Frank R. Blattner

Supplementary Online Materials

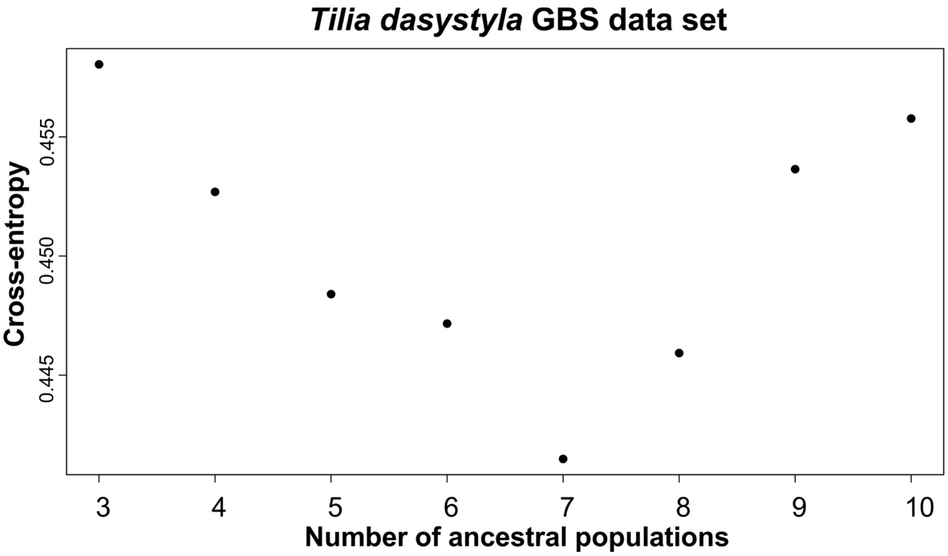

Fig. S1 Cross-entropy evaluation to arrive at an optimal number for K in population assignment analysis. Cross-entropy values are plotted for K = 3 to 10 with a lowest value at K = 7.

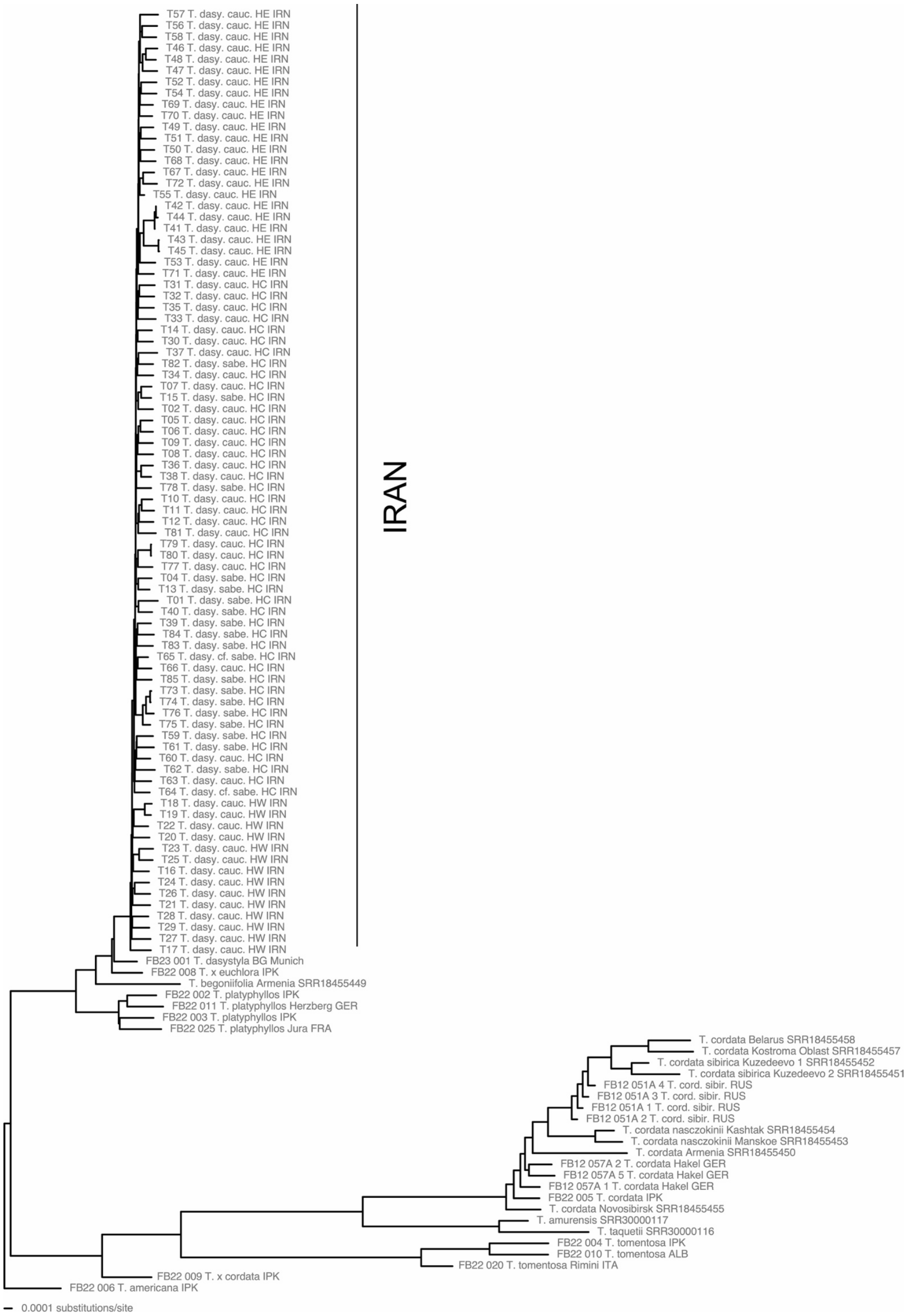

Fig. S2 Neighbor-joining tree for initial inference of genetic distances for the individuals in the GBS analyses of *Tilia*. The tree indicates that all Iranian individuals in the analysis form a genetically narrow and monophyletic group.

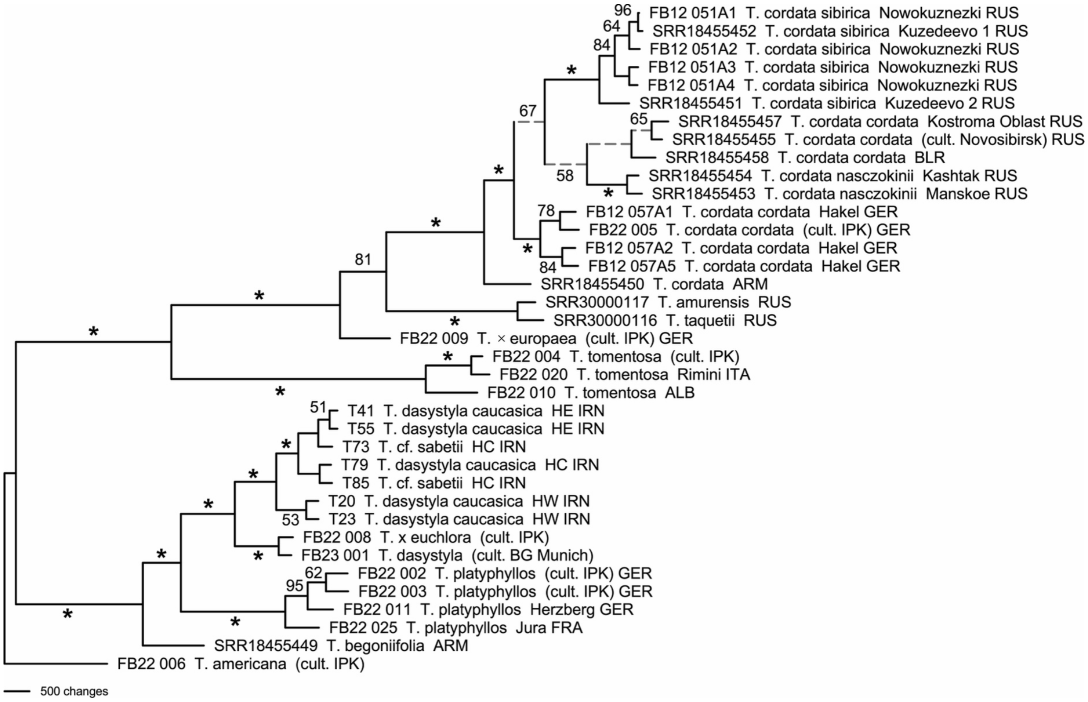

Fig. S3 Phylogenetic analysis of the GBS-derived "species" dataset by MP resulted in two equally parsimonious trees. Branches collapsing in the strict consensus tree are provided as dashed gray lines. Numbers along branches are bootstrap values (%) derived from 1000 bootstrap re-samples, with asterisks indicating 100% support. The tree length is 52,780 steps (CI = 0.68, RI = 0.86).

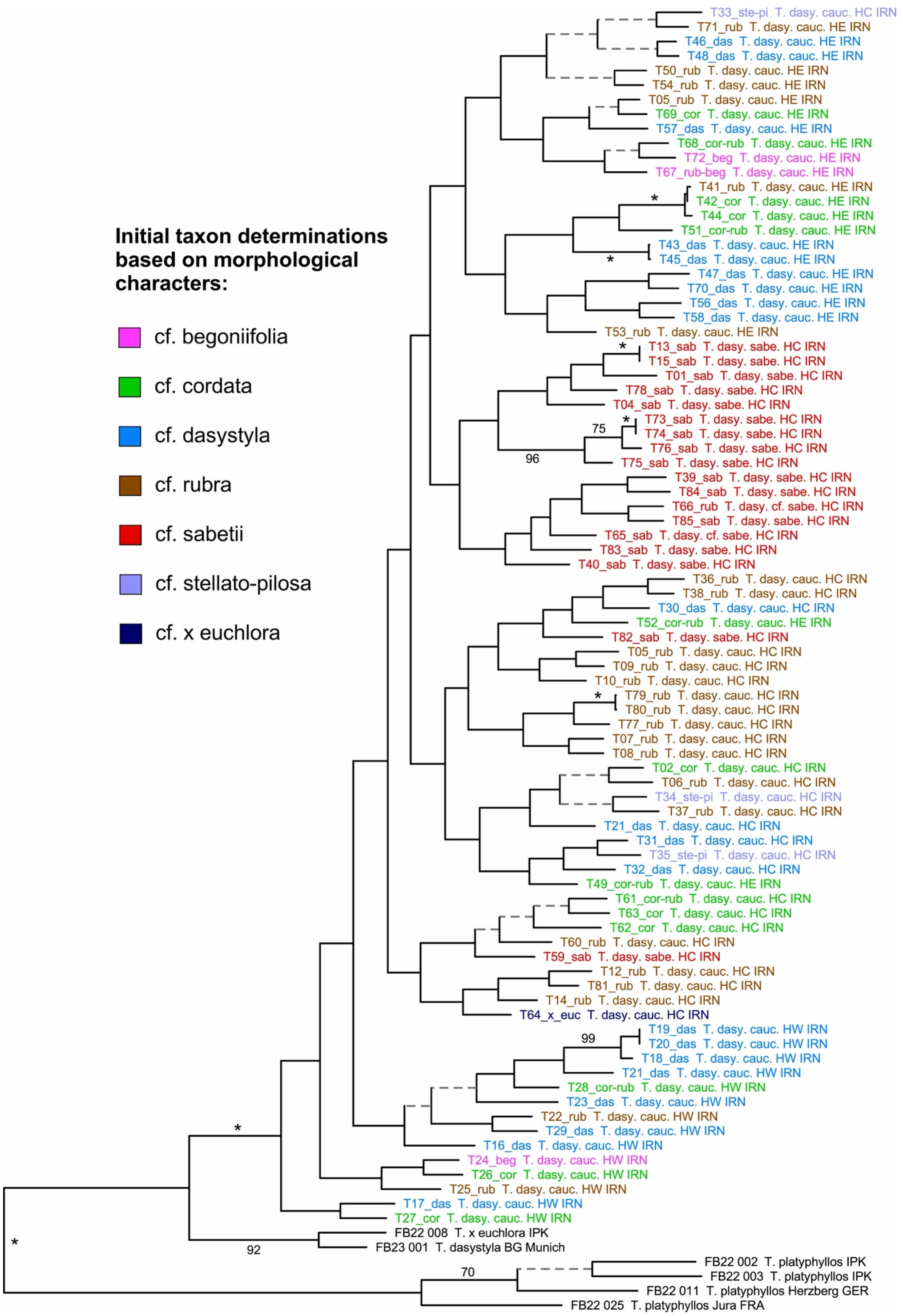

Fig. S4 Initial species determinations (abbreviated after individual numbers and color coded) for the Hyrcanian *Tilia* individuals plotted on the parsimony tree of Figure 1 showing that none of the taxa form a monophyletic group.

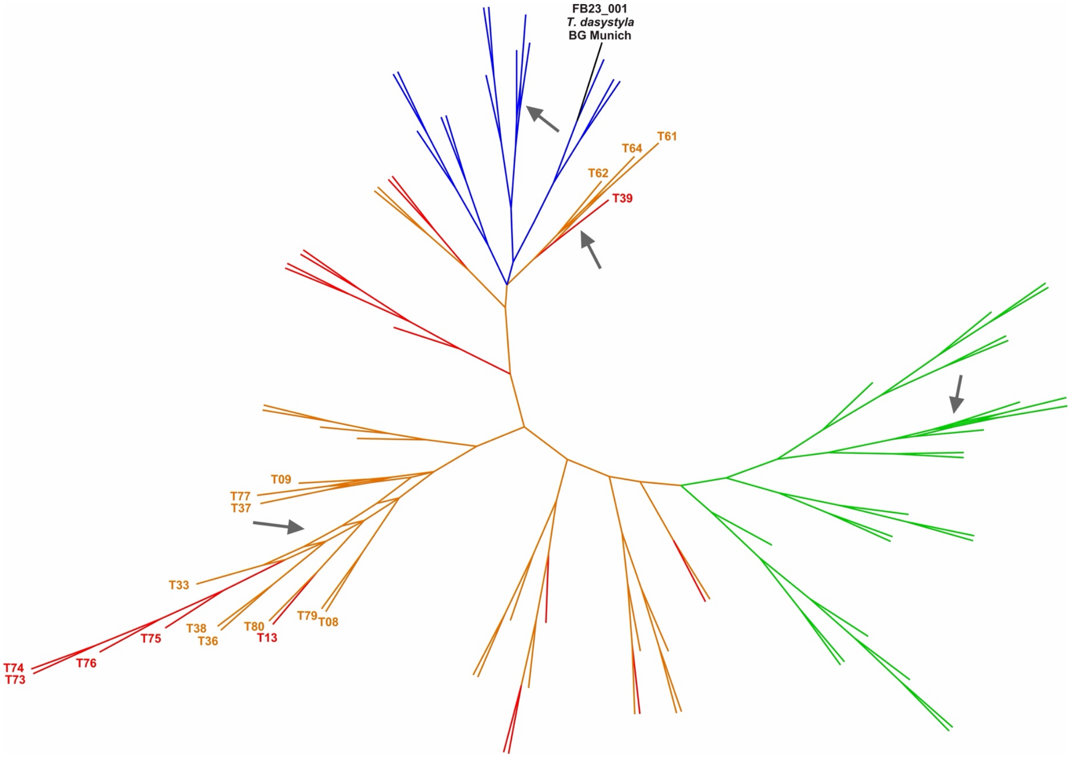

Fig. S5 Consensus network based on 750 ML trees of Hyrcanian *Tilia dasystyla* populations. An individual from the Botanical Garden Munich was included as outgroup. Colors code the geographic regions with western populations (blue), central Hyrcanian populations (orange), and eastern populations (green). Red color indicates cf. *sabetii* individuals occurring within the central Hyrcanian Forest stands. Arrows depict the four parts where reticulate relationships were inferred, although overall reticulation is low and relationships are mostly tree-like. Numbers of individuals (Supplementary Table S1) are provided for reticulate structures in mixed clusters of *cf. sabetii* and central Hyrcanian subsp. *caucasica* individuals.

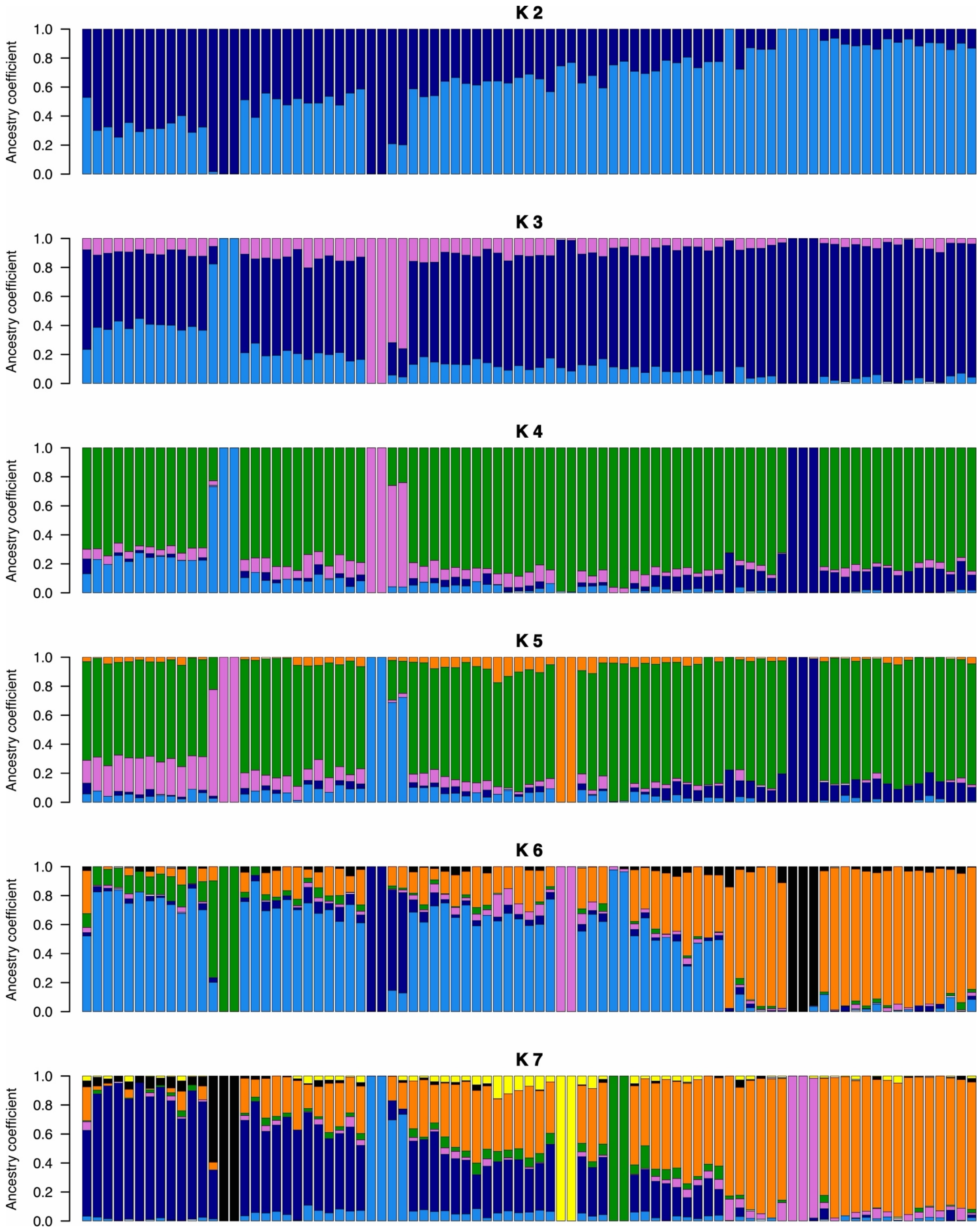

Fig. S6 Bayesian population assignment analyses at K = 2 to K = 7. The individuals are ordered longitudinal, with the individuals from the west on the left and the eastern individuals to the right. Hierarchical substructuring is weak in the data and the west to east pattern of colonization becomes only clear at K = 6 and K = 7.

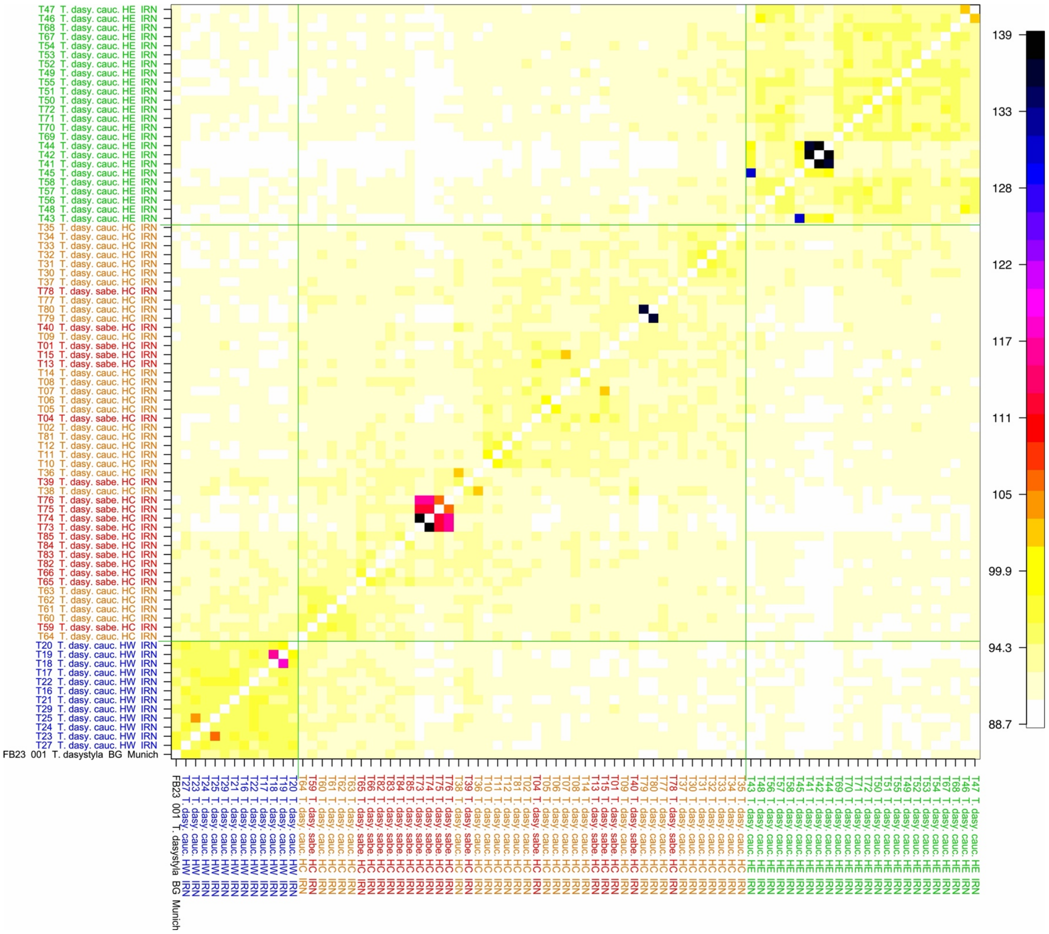

Fig. S7 Simple coancestry matrix derived from an analysis of the GBS data of the Iranian *Tilia* individuals visualized in FINERADSTRUCTURE. Individuals were ordered longitudinally. The plot shows that differentiation between individuals in Iran is generally low and that cf. *sabetii* individuals do not form a genetic group of their own. Colors of labels correspond to sampling regions: blue = western Hyrcanian (HW IRN), orange/red = central Hyrcanian (HC IRN), and green = eastern Hyrcanian (HE IRN), while red indicates individuals determined as cf. *sabetii*.

Table S1 *Tilia* materials analyzed in this study. Herbaria are abbreviated according to Index Herbariorum as GAT (Institute of Plant Genetics and Crop Plant Research, IPK Gatersleben, Germany), OSBU (Herbarium of the Botanical Institute of the University of Osnabrück, Germany), M (Botanische Staatssamlung und Botanischer Garten München, Munich, Germany) and HUI (Herbarium of the University of Isfahan, Iran).

Code	Taxon	Taxon according to Flora of Iran (Zare, 2017)	Locality	Geographic coordinates	Eleva-tion	Date	Herbarium voucher	Genome size 2C (pg)	Ploidy level	Taxon determination
FB22_002	*T. platyphyllos*	-	Cultivated: Germany, IPK Gatersleben, W of Genetics Bld.; B 7	51.827881°N, 011.275636°E	112 m	7.6.2022	GAT 102029	1.97	2*x*	F.R. Blattner
FB22_003	*T. platyphyllos*	-	Cultivated: Germany, IPK Gatersleben, Bot. Garden, large meadow; S 39	51.823616°N, 011.278475°E	112 m	7.6.2022	GAT 102031	-	-	F.R. Blattner
FB22_004	*T. tomentosa*	-	Unknown; cultivated: Germany, IPK Gatersleben, Bot. Garden, lilac hedge; S 15.2	51.823972°N, 011.280052°E	112 m	7.6.2022	GAT 110637/ 110638	2.18	2*x*	F.R. Blattner
FB22_005	*T. cordata* subsp. *cordata*	-	Cultivated: Germany, IPK Gatersleben, Bot. Garden, forest; S 26	51.824165°N, 011.279013°E	112 m	7.6.2022	GAT 102032 / 102033	2.03	2*x*	F.R. Blattner
FB22_006	*T. americana*	-	Unknown; cultivated: Germany, IPK Gatersleben, W of Genetics Bld.; B 5.2	51.828022°N, 011.274571°E	112 m	7.6.2022	GAT 102034	2.21	2*x*	F.R. Blattner
FB22_008	*T.* × *euchlora*	-	Unknown; cultivated: Germany, IPK Gatersleben, N of lecture hall at fence; D 3.0	51.827368°N, 011.278998°E	112 m	7.6.2022	GAT 102035	4.09	4*x*	F.R. Blattner
FB22_009	*T.* × *europaea*	-	Cultivated: Germany, IPK Gatersleben, S of Heating Station; 17.0	51.824620°N, 011.277316°E	112 m	7.6.2022	GAT 102036	1.99	2*x*	F.R. Blattner
FB22_010	*T. tomentosa*	-	NE Albania, Tropoje, c. 10 km N of Viçidol	42.44001°N, 020.14907°E	700 m	3.6.2022	GAT 102037 / 102038	-	-	F.R. Blattner
FB22_011	*T. platyphyllos*	-	Germany, Herzberg/ Harz; large tree in front of castle, with sign naming it (wrongly) "Conrads Linde"	51.655177°N, 010.329826°E	272 m	12.6.2022	GAT 102039	-	-	F.R. Blattner
FB22_020	*T. tomentosa*	-	Italy, Rimini, Sant' Antimo; roadside	43.80832°N, 012.19737°E	417 m	26.6.2022	GAT 102040	-	-	F.R. Blattner
FB22_025	*T. platyphyllos*	-	France, Jura, Le Frasnois/Lac de Narley; old tree within campground	46.640417°N, 005.908274°E	758 m	27.7.2022	GAT 102041	-	-	F.R. Blattner
FB12_057_ A1,2,5	*T. cordata* subsp. *cordata*	-	Germany, Hakel (natural mixed linden forest) N of Gatersleben	51.8638°N, 011.3154°E	150 m	23.8.2012	s.n.	-	-	F.R. Blattner
FB12_051_ A1,2,3,4	*T. cordata* subsp. *sibirica*	-	Russia, S Siberia, Nowokusnezki rajon, Kemerowo Oblast; c. 50 km S of Novokushnetsk on slopes above Reka Bol'shoye Tesh	53.32136°N, 087.25422°E	380 m	7.8.2012	OSBU 22119	-	-	F.R. Blattner
FB23_001	*T. dasystyla*	-	Unknown; cultivated: Bot. Garten München-Nymphenburg	–	–	29.6.2023	M 2280	4.2	4*x*	S. Pfanzelt
T01	*T. dasystyla* cf. *sabetii*	*T. sabetii*	Iran, Mazandaran, Chamestan, Tisheh forest	36.475176°N, 052.100465°E	337 m	8.6.2022	HUI N.Ala-25742		4*x*	N. Ala, H. Zare
T02	*T. dasystyla* subsp. *caucasica*	*T.* cf. *cordata*	Iran, Mazandaran, Noor, Anarjar	36.513768°N, 051.985778°E	184 m	13.6.2022	HUI N.Ala-25743		4*x*	N. Ala, H. Zare

Code	Taxon	Taxon according to Flora of Iran (Zare, 2017)	Locality	Geographic coordinates	Eleva-tion	Date	Herbarium voucher	Genome size 2C (pg)	Ploidy level	Taxon determination
T03	*T. dasystyla* subsp. *caucasica*	*T.* cf. *cordata*	Iran, Mazandaran, Noor, Anarjar	36.513768°N, 051.985778°E	184 m	13.6.2022	HUI N.Ala-25744		4*x*	N. Ala, H. Zare
T04	*T. dasystyla* cf. *sabetii*	*T. sabetii*	Iran, Mazandaran, Noor, Anarjar	36.513768°N, 051.985778°E	220 m	13.6.2022	HUI N.Ala 25745	4.76	4*x*	N. Ala, H. Zare
T05	*T. dasystyla* subsp. *caucasica*	*T.* cf. *rubra*	Iran, Mazandaran, Noor, Anarjar	36.513768°N, 051.985778°E	167 m	13.6.2022	HUI N.Ala-25746		4*x*	N. Ala, H. Zare
T06	*T. dasystyla* subsp. *caucasica*	*T.* cf. *rubra*	Iran, Mazandaran, Noor, Anarjar	36.513768°N, 051.985778°E	200 m	13.6.2022	HUI N.Ala-25747		4*x*	N. Ala, H. Zare
T07	*T. dasystyla* subsp. *caucasica*	*T.* cf. *rubra*	Iran, Mazandaran, Noor, Anarjar	36.513768°N, 051.985778°E	138 m	13.6.2022	HUI N.Ala-25748		4*x*	N. Ala, H. Zare
T08	*T. dasystyla* subsp. *caucasica*	*T.* cf. *rubra*	Iran, Mazandaran, Noor, Anarjar	36.513768°N, 051.985778°E	216 m	13.6.2022	HUI N.Ala-25749		4*x*	N. Ala, H. Zare
T09	*T. dasystyla* subsp. *caucasica*	*T.* cf. *rubra*	Iran, Mazandaran, Chamestan, Tisheh	36.474871°N, 052.100833°E	337 m	8.6.2022	HUI N.Ala-25750		4*x*	N. Ala, H. Zare
T10	*T. dasystyla* subsp. *caucasica*	*T. rubra*	Iran, Mazandaran, Royan, Abpari waterfall	36.479193°N, 051.916019°E	231 m	8.6.2022	HUI N.Ala-25751		4*x*	N. Ala, H. Zare
T11	*T. dasystyla* subsp. *caucasica*	*T. rubra*	Iran, Mazandaran, Royan, Abpari waterfall	36.479193°N, 051.916019°E	227 m	8.6.2022	HUI N.Ala-25752		4*x*	N. Ala, H. Zare
T12	*T. dasystyla* subsp. *caucasica*	*T. rubra*	Iran, Mazandaran, Royan, Abpari waterfall	36.480324°N, 051.916910°E	226 m	8.6.2022	HUI N.Ala-25753		4*x*	N. Ala, H. Zare
T13	*T. dasystyla* cf. *sabetii*	*T. sabetii*	Iran, Mazandaran, Noor, Anarjar	36.513768°N, 051.985778°E	199 m	13.6.2022	HUI N.Ala-25754		4*x*	N. Ala, H. Zare
T14	*T. dasystyla* subsp. *caucasica*	*T.* cf. *rubra*	Iran, Mazandaran, Noor, Anarjar	36.513768°N, 051.985778°E	216 m	13.6.2022	HUI N.Ala-25755		4*x*	N. Ala, H. Zare
T15	*T. dasystyla* cf. *sabetii*	*T. sabetii*	Iran, Mazandaran, Noor, Anarjar	36.513768°N, 051.985778°E	201 m	13.6.2022	HUI N.Ala-25756		4*x*	N. Ala, H. Zare
T16	*T. dasystyla* subsp. *caucasica*	*T.* cf. *dasystyla*	Iran, Guilan, Asalem, Tazehabad	37.806847°N, 048.942280°E	106 m	5.7.2022	HUI N.Ala-25757		4*x*	N. Ala, H. Zare
T17	*T. dasystyla* subsp. *caucasica*	*T.* cf. *dasystyla*	Iran, Guilan, Asalem, Tazehabad	37.806847°N, 048.942280°E	149 m	5.7.2022	HUI N.Ala-25758		4*x*	N. Ala, H. Zare
T18	*T. dasystyla* subsp. *caucasica*	*T.* cf. *dasystyla*	Iran, Guilan, Asalem, Kishkhaleh	37.643866°N, 049.036981°E	656 m	5.7.2022	HUI N.Ala-25759		4*x*	N. Ala, H. Zare
T19	*T. dasystyla* subsp. *caucasica*	*T.* cf. *dasystyla*	Iran, Guilan, Asalem, Kishkhaleh	37.643866°N, 049.036981°E	606 m	5.7.2022	HUI N.Ala-25760		4*x*	N. Ala, H. Zare
T20	*T. dasystyla* subsp. *caucasica*	*T. dasystyla*	Iran, Guilan, Asalem, Kishkhaleh	37.643866°N, 049.036981°E	606 m	5.7.2022	HUI N.Ala-25761		4*x*	N. Ala, H. Zare
T21	*T. dasystyla* subsp. *caucasica*	*T.* cf. *dasystyla*	Iran, Guilan, Talesh, Kharchgil	37.695445°N, 048.916497°E	149 m	5.7.2022	HUI N.Ala 25762	4.70	4*x*	N. Ala, H. Zare

Code	Taxon	Taxon according to Flora of Iran (Zare, 2017)	Locality	Geographic coordinates	Eleva-tion	Date	Herbarium voucher	Genome size 2C (pg)	Ploidy level	Taxon determination
T22	*T. dasystyla* subsp. *caucasica*	*T. rubra*	Iran, Guilan, Asalem	37.728798°N, 048.958248°E	51 m	5.7.2022	HUI N.Ala 25763	4.46	4*x*	N. Ala, H. Zare
T23	*T. dasystyla* subsp. *caucasica*	*T.* cf. *dasystyla*	Iran, Guilan, Hashtpar, Kishoonbon	37.797273°N, 048.835507°E	293 m	5.7.2022	HUI N.Ala-25764		4*x*	N. Ala, H. Zare
T24	*T. dasystyla* subsp. *caucasica*	*T.* cf. *begoniifolia*	Iran, Guilan, Hashtpar, Kishoonbon	37.797273°N, 048.835507°E	314 m	5.7.2022	HUI N.Ala 25765	4.92	4*x*	N. Ala, H. Zare
T25	*T. dasystyla* subsp. *caucasica*	*T. rubra*	Iran, Guilan, Hashtpar, Kishoonbon	37.797273°N, 048.835507°E	278 m	5.7.2022	HUI N.Ala-25766		4*x*	N. Ala, H. Zare
T26	*T. dasystyla* subsp. *caucasica*	*T. cordata*	Iran, Guilan, Hashtpar, Rick valley	37.751043°N, 048.693399°E	144 m	5.7.2022	HUI N.Ala 25767	4.78	4*x*	N. Ala, H. Zare
T27	*T. dasystyla* subsp. *caucasica*	*T. cordata*	Iran, Guilan, Hashtpar, Rick valley	37.751043°N, 048.693399°E	150 m	5.7.2022	HUI N.Ala 25768	4.85	4*x*	N. Ala, H. Zare
T28	*T. dasystyla* subsp. *caucasica*	*T.* cf. *cordata* or *rubra*	Iran, Guilan, Asalem, Gijav	37.697706°N, 048.870330°E	293 m	5.7.2022	HUI N.Ala-25769		4*x*	N. Ala, H. Zare
T29	*T. dasystyla* subsp. *caucasica*	*T.* cf. *dasystyla*	Iran, Guilan, Asalem, Gijav	37.697706°N, 048.870330°E	293 m	5.7.2022	HUI N.Ala 25770	4.72	4*x*	N. Ala, H. Zare
T30	*T. dasystyla* subsp. *caucasica*	*T.* cf. *dasystyla*	Iran, Mazandaran, Sari, Partkola	36.153002°N, 053.361777°Em	1307	10.7.2022	HUI N.Ala-25771		4*x*	N. Ala, H. Zare
T31	*T. dasystyla* subsp. *caucasica*	*T.* cf. *dasystyla*	Iran, Mazandaran, Sari, Partkola	36.153002°N, 053.361777°Em	1335	10.7.2022	HUI N.Ala-25772		4*x*	N. Ala, H. Zare
T32	*T. dasystyla* subsp. *caucasica*	*T.* cf. *dasystyla*	Iran, Mazandaran, Sari, Partkola	36.153002°N, 053.361777°Em	1400	10.7.2022	HUI N.Ala-25773		4*x*	N. Ala, H. Zare
T33	*T. dasystyla* subsp. *caucasica*	*T.* cf. *stellato-pilosa*	Iran, Mazandaran, Sari, Partkola	36.153002°N, 053.361777°Em	1326	10.7.2022	HUI N.Ala 25774	4.93	4*x*	N. Ala, H. Zare
T34	*T. dasystyla* subsp. *caucasica*	*T.* cf. *stellato-pilosa*	Iran, Mazandaran, Sari, Partkola, Ajgheh	36.153002°N, 053.361777°E	936 m	10.7.2022	HUI N.Ala 25775	4.82	4*x*	N. Ala, H. Zare
T35	*T. dasystyla* subsp. *caucasica*	*T.* cf. *stellato-pilosa*	Iran, Mazandaran, Sari, Partkola	36.153002°N, 053.361777°Em	1334	10.7.2022	HUI N.Ala-25776		4*x*	N. Ala, H. Zare
T36	*T. dasystyla* subsp. *caucasica*	*T. rubra*	Iran, Mazandaran, Nowshahr, Chalandar	36.566797°N, 051.693028°E	88 m	8.6.2022	HUI N.Ala 25777	5.01	4*x*	N. Ala, H. Zare
T37	*T. dasystyla* subsp. *caucasica*	*T. rubra*	Iran, Mazandaran, Savadkooh, Shirgah, Palang darreh	36.278866°N, 052.938726°E	460 m	16.7.2022	HUI N.Ala-25778		4*x*	N. Ala, H. Zare
T38	*T. dasystyla* subsp. *caucasica*	*T. rubra*	Iran, Mazandaran, Nowshahr, Chalandar	36.566797°N, 051.693028°E	93 m	8.6.2022	HUI N.Ala-25779		4*x*	N. Ala, H. Zare
T39	*T. dasystyla* cf. *sabetii*	*T. sabetii*	Iran, Mazandaran, Nowshahr, Chalandar	36.566797°N, 051.693028°E	102 m	8.6.2022	HUI N.Ala-25780		4*x*	N. Ala, H. Zare

Code	Taxon	Taxon according to Flora of Iran (Zare, 2017)	Locality	Geographic coordinates	Eleva-tion	Date	Herbarium voucher	Genome size 2C (pg)	Ploidy level	Taxon determination
T40	*T. dasystyla* cf. *sabetii*	*T. sabetii*	Iran, Mazandaran, Chamestan, Tisheh	36.475176°N, 052.100465°E	295 m	15.6.2022	HUI N.Ala-25781	4.75	4*x*	N. Ala, H. Zare
T41	*T. dasystyla* subsp. *caucasica*	*T.* cf. *rubra*	Iran, Golestan, Gorgan, Shamooshak	36.734946°N, 054.280292°E	862 m	20.7.2022	HUI N.Ala-25782		4*x*	N. Ala, H. Zare
T42	*T. dasystyla* subsp. *caucasica*	*T.* cf. *cordata*	Iran, Golestan, Gorgan, Shamooshak	36.734946°N, 054.280292°E	862 m	20.7.2022	HUI N.Ala-25783		4*x*	N. Ala, H. Zare
T43	*T. dasystyla* subsp. *caucasica*	*T.* cf. *dasystyla*	Iran, Golestan, Galikesh, Loweh	37.354396°N, 055.666033°E	959 m	20.7.2022	HUI N.Ala-25784		4*x*	N. Ala, H. Zare
T44	*T. dasystyla* subsp. *caucasica*	*T.* cf. *cordata*	Iran, Golestan, Gorgan, Shamooshak	36.734946°N, 054.280292°E	859 m	20.7.2022	HUI N.Ala-25785	4.74	4*x*	N. Ala, H. Zare
T45	*T. dasystyla* subsp. *caucasica*	*T.* cf. *dasystyla*	Iran, Golestan, Gorgan, Shamooshak	36.734946°N, 054.280292°E	859 m	20.7.2022	HUI N.Ala-25786		4*x*	N. Ala, H. Zare
T46	*T. dasystyla* subsp. *caucasica*	*T.* cf. *dasystyla*	Iran, Golestan, Galikesh, Loweh	37.354396°N, 055.666033°E	859 m	20.7.2022	HUI N.Ala-25787	5.31	4*x*	N. Ala, H. Zare
T47	*T. dasystyla* subsp. *caucasica*	*T.* cf. *dasystyla*	Iran, Golestan, Galikesh, Loweh	37.354396°N, 055.666033°E	862 m	20.7.2022	HUI N.Ala-25788		4*x*	N. Ala, H. Zare
T48	*T. dasystyla* subsp. *caucasica*	*T.* cf. *dasystyla*	Iran, Golestan, Galikesh, Loweh	37.354396°N, 055.666033°E	859 m	20.7.2022	HUI N.Ala-25789		4*x*	N. Ala, H. Zare
T49	*T. dasystyla* subsp. *caucasica*	*T.* cf. *cordata* or *rubra*	Iran, Golestan, Aliabadkatool, Afratakhteh road	36.800591°N, 054.972139°E	668 m	20.7.2022	HUI N.Ala-25790	5.06	4*x*	N. Ala, H. Zare
T50	*T. dasystyla* subsp. *caucasica*	*T.* cf. *rubra*	Iran, Golestan, Aliabad, Zarringol	36.880423°N, 054.960030°E	492 m	20.7.2022	HUI N.Ala-25791		4*x*	N. Ala, H. Zare
T51	*T. dasystyla* subsp. *caucasica*	*T.* cf. *cordata* or *rubra*	Iran, Golestan, Aliabadkatool, Afratakhteh road	36.800591°N, 054.972139°E	960 m	20.7.2022	HUI N.Ala-25792	5.10	4*x*	N. Ala, H. Zare
T52	*T. dasystyla* subsp. *caucasica*	*T.* cf. *cordata* or *rubra*	Iran, Golestan, Aliabadkatool, Afratakhteh road	36.800591°N, 054.972139°E	945 m	20.7.2022	HUI N.Ala-25793		4*x*	N. Ala, H. Zare
T53	*T. dasystyla* subsp. *caucasica*	*T. rubra*	Iran, Golestan, Aliabadkatool, Afratakhteh road	36.800591°N, 054.972139°E	954 m	20.7.2022	HUI N.Ala-25794	4.67	4*x*	N. Ala, H. Zare
T54	*T. dasystyla* subsp. *caucasica*	*T. rubra*	Iran, Golestan, Aliabadkatool, Afratakhteh road	36.800591°N, 054.972139°Em	1057	20.7.2022	HUI N.Ala-25795		4*x*	N. Ala, H. Zare
T55	*T. dasystyla subsp. caucasica*	*T. dasystyla*	Iran, Golestan, Galikesh, Loweh	37.354396°N, 055.666033°E	859 m	20.7.2022	HUI N.Ala-25796		4*x*	N. Ala, H. Zare

**Code**	**Taxon**	**Taxon according to Flora of Iran (Zare, 2017)**	**Locality**	**Geographic coordinates**	**Eleva-tion**	**Date**	**Herbarium voucher**	**Genome size 2C (pg)**	**Ploidy level**	**Taxon determination**
T56	*T. dasystyla* subsp. *caucasica*	*T. dasystyla*	Iran, Golestan, Galikesh, Loweh	37.354396°N, 055.666033°E	1265 m	20.7.2022	HUI N.Ala-25797	4.72	4*x*	N. Ala, H. Zare
T57	*T. dasystyla* subsp. *caucasica*	*T. dasystyla*	Iran, Golestan, Galikesh, Loweh	37.354396°N, 055.666033°E	1290 m	20.7.2022	HUI N.Ala-25798		4*x*	N. Ala, H. Zare
T58	*T. dasystyla* subsp. *caucasica*	*T. dasystyla*	Iran, Golestan, Galikesh, Loweh	37.354396°N, 055.666033°E	1345 . m	20.7.2022	HUI N.Ala-25799		4*x*	N. Ala, H. Zare
T59	*T. dasystyla* cf. *sabetii*	*T. sabetii*	Iran, Mazandaran, Chalus, Kandovan, Dozdband	36.337577°N, 051.256809°E	1786 m	15.8.2022	HUI N.Ala-25800	4.79	4*x*	N. Ala, H. Zare
T60	*T. dasystyla* subsp. *caucasica*	*T. rubra*	Iran, Mazandaran, Chalus, Kandovan, Dozdband	36.337577°N, 051.256809°E	1753 m	15.8.2022	HUI N.Ala-25801		4*x*	N. Ala, H. Zare
T61	*T. dasystyla* subsp. *caucasica*	*T.* cf. *cordata* or *rubra*	Iran, Mazandaran, Chalus, Kandovan road, Dozdband	36.333334°N, 051.256113°E	1753 m	15.8.2022	HUI N.Ala-25802	4.71	4*x*	N. Ala, H. Zare
T62	*T. dasystyla* subsp. *caucasica*	*T. cordata*	Iran, Mazandaran, Chalus, Kandovan, Dozdband	36.333334°N, 051.256113°E	1804 m	15.8.2022	HUI N.Ala-25803	4.99	4*x*	N. Ala, H. Zare
T63	*T. dasystyla* subsp. *caucasica*	*T. cordata*	Iran, Mazandaran, Chalus, Kandovan road	36.264518°N, 051.261004°E	1076 m	15.8.2022	HUI N.Ala-25804		4*x*	N. Ala, H. Zare
T64	*T. dasystyla* subsp. *caucasica*	*T.* × *euchlora*	Iran, Mazandaran, Chalus, Kandovan road, Dozdband	36.264689°N, 051.258986°E	1338 m	15.8.2022	HUI N.Ala-25805	4.71	4*x*	N. Ala, H. Zare
T65	*T. dasystyla* cf. *sabetii*	*T. sabetii*	Iran, Mazandaran, Nowshahr, Najjardeh, Kheyroodkenar, Protected forest	36.605031°N, 051.558471°E	28 m	10.8.2022	HUI N.Ala-25806		4*x*	N. Ala, H. Zare
T66	*T. dasystyla* cf. *sabetii*	*T. sabetii*	Iran, Mazandaran, Nowshahr, Najjardeh, Kheyroodkenar, Protected forest	36.605031°N, 051.558471°E	28 m	10.8.2022	HUI N.Ala-25807		4*x*	N. Ala, H. Zare
T67	*T. dasystyla* subsp. *caucasica*	*T.* cf. *rubra* or *begoniifolia*	Iran, Golestan, Ramian, Paqaleh	36.894822°N, 055.085857°E	1430 m	15.8.2022	HUI N.Ala-25808	4.76	4*x*	N. Ala, H. Zare
T68	*T. dasystyla* subsp. *caucasica*	*T.* cf. *rubra* or *cordata*	Iran, Golestan, Ramian, Paqaleh	36.894822°N, 055.085857°E	1462 m	15.8.2022	HUI N.Ala-25809		4*x*	N. Ala, H. Zare
T69	*T. dasystyla* subsp. *caucasica*	*T. cordata*	Iran, Golestan, Aliabadkatool, after Zarringol, Doupol	36.883919°N, 054.967629°E	1051 m	15.8.2022	HUI N.Ala-25810	4.72	4*x*	N. Ala, H. Zare

Code	Taxon	Taxon according to Flora of Iran (Zare, 2017)	Locality	Geographic coordinates	Elevation	Date	Herbarium voucher	Genome size 2C (pg)	Ploidy level	Taxon determination
T70	*T. dasystyla* subsp. *caucasica*	*T. dasystyla*	Iran, Golestan, Aliabadkatool, after Zarringol, Doupol	36.883919°N, 054.967629°E	1072 m	15.8.2022	HUI N.Ala 25811	4.91	4*x*	N. Ala, H. Zare
T71	*T. dasystyla* subsp. *caucasica*	*T. rubra*	Iran, Golestan, Aliabadkatool, after Zarringol, Doupol	36.883919°N, 054.967629°E	1263 m	15.8.2022	HUI N.Ala 25812	4.74	4*x*	N. Ala, H. Zare
T72	*T. dasystyla* subsp. *caucasica*	*T. begoniifolia*	Iran, Golestan, Aliabadkatool, after Zarringol, Doupol	36.883919°N, 054.967629°E	1263 m	15.8.2022	HUI N.Ala 25813	4.81	4*x*	N. Ala, H. Zare
T73	*T. dasystyla* cf. *sabetii*	*T. sabetii*	Iran, Mazandaran, Nowshahr, Chelak	36.587974°N, 051.632214°E	74 m	4.9.2022	HUI N.Ala-25814		4*x*	N. Ala, H. Zare
T74	*T. dasystyla* cf. *sabetii*	*T. sabetii*	Iran, Mazandaran, Nowshahr, Chelak	36.587974°N, 051.632214°E	74 m	4.9.2022	HUI N.Ala-25815		4*x*	N. Ala, H. Zare
T75	*T. dasystyla* cf. *sabetii*	*T. sabetii*	Iran, Mazandaran, Nowshahr, Chelak	36.587974°N, 051.632214°E	64 m	4.9.2022	HUI N.Ala-25816		4*x*	N. Ala, H. Zare
T76	*T. dasystyla* cf. *sabetii*	*T. sabetii*	Iran, Mazandaran, Nowshahr, Chelak	36.588469°N, 051.632435°E	40 m	4.9.2022	HUI N.Ala-25817		4*x*	N. Ala, H. Zare
T77	*T. dasystyla* subsp. *caucasica*	*T. rubra*	Iran, Mazandaran, Chamestan, Vaz	36.321061°N, 052.124288°E	1382 m	8.9.2022	HUI N.Ala-25818		4*x*	N. Ala, H. Zare
T78	*T. dasystyla* cf. *sabetii*	*T. sabetii*	Iran, Mazandaran, Chamestan, Vaz	36.317945°N, 052.126614°E	1382 m	8.9.2022	HUI N.Ala 25819	4.68	4*x*	N. Ala, H. Zare
T79	*T. dasystyla* subsp. *caucasica*	*T. rubra*	Iran, Mazandaran, Chamestan, Vaz	36.314867°N, 052.121405°E	1447 m	8.9.2022	HUI N.Ala-25820		4*x*	N. Ala, H. Zare
T80	*T. dasystyla* subsp. *caucasica*	*T. rubra*	Iran, Mazandaran, Chamestan, Vaz	36.313096°N, 052.120931°E	1834 m	8.9.2022	HUI N.Ala-25821		4*x*	N. Ala, H. Zare
T81	*T. dasystyla* subsp. *caucasica*	*T. rubra*	Iran, Mazandaran, Royan, Abpari waterfall	36.480324°N, 051.916910°E	1834 m	20.6.2022	HUI N.Ala-25822		4*x*	N. Ala, H. Zare
T82	*T. dasystyla* cf. *sabetii*	*T. sabetii*	Iran, Mazandaran, Nowshahr, Najjardeh, Kheyroodkenar, Protected forest	36.600356°N, 051.555360°E	49 m	15.9.2022	HUI N.Ala-25823		4*x*	N. Ala, H. Zare
T83	*T. dasystyla* cf. *sabetii*	*T. sabetii*	Iran, Mazandaran, Nowshahr, Najjardeh, Kheyroodkenar, Protected forest	36.600356°N, 051.555360°E	49 m	15.9.2022	HUI N.Ala-25824		4*x*	N. Ala, H. Zare
T84	*T. dasystyla* cf. *sabetii*	*T. sabetii*	Iran, Mazandaran, Nowshahr, Najjardeh, Kheyroodkenar, Protected forest	36.585372°N, 051.553913°E	132 m	15.9.2022	HUI N.Ala-25825		4*x*	N. Ala, H. Zare

Code	Taxon	Taxon according to Flora of Iran (Zare, 2017)	Locality	Geographic coordinates	Eleva-tion	Date	Herbarium voucher	Genome size 2C (pg)	Ploidy level	Taxon determination
T85	*T. dasystyla* cf. *sabetii*	*T. sabetii*	Iran, Mazandaran, Nowshahr, Kheyroodkenar, Protected forest	36.585372°N, 051.553913°E	138 m	15.9.2022	HUI N.Ala 25826	4.73	4*x*	N. Ala, H. Zare
SRR31000117	*T. amurensis*	-	Russia, Khabarovskiy kray, Khabarovsk District	48.88°N 134.76°E	-	-	-	-	-	Shekhovtsov et al. (2022)
SRR18455449	*T. begoniifolia* [≠ *T. dasystyla* subsp. *caucasica* (V.ENGL.) PIGOTT]	-	Armenia, Tavesh Province, Gosh village	40.729°N 45.002°E	-	-	-	-	-	Shekhovtsov et al. (2022)
SRR18455450	*T. cordata*	-	Armenia, Lori Province, Chkalov settlement	40.928°N 44.626°E	-	-	-	-	-	Shekhovtsov et al. (2022)
SRR18455455	*T. cordata* subsp. *cordata*	-	Russia, cultivated in Novosibirsk	54.84788°N 83.10825°E	-	-	-	-	-	Shekhovtsov et al. (2022)
SRR18455457	*T. cordata* subsp. *cordata*	-	Russia, Kostroma Oblast, Sledovo village	57.76333°N 41.25183°E	137 m	-	-	-	-	Shekhovtsov et al. (2022)
SRR18455458	*T. cordata* subsp. *cordata*	-	Belarus, Krichev Natural Reserve	53.643°N 31.883°E	-	-	-	-	-	Shekhovtsov et al. (2022)
SRR18455453	*T. cordata* subsp. *nasczokinii*	-	Russia, Mana Village, Manskoe	55.9643 N 92.5029°E	-	-	-	-	-	Shekhovtsov et al. (2022)
SRR18455454	*T. cordata* subsp. *nasczokinii*	-	Russia, Kashtak Valley	55.9575 N 92.7793°E	-	-	-	-	-	Shekhovtsov et al. (2022)
SRR18455452	*T. cordata* subsp. *sibirica*	-	Russia, Kuzedeevo	53.3281°N 87.2806°E	-	-	-	-	-	Shekhovtsov et al. (2022)
SRR18455451	*T. cordata* subsp. *sibirica*	-	Russia, Kuzedeevo	53.3281°N 87.2806°E	-	-	-	-	-	Shekhovtsov et al. (2022)
SRR31000116	*T. taquetii* [possibly *T. amurensis* subsp. *taquetii* (C.K. SCHNEID.) PIGOTT]	-	Russia, Far East	-	-	-	-	-	-	Shekhovtsov et al. (2022)

Table S2 Analysis of heterozygosity to discern di- and tetraploidy in the set of Iranian individuals

Individual	Observed het. positions	Sites	NetSites	NLoci	Fraction of heterozygous sites
FB22_002 T. platyphyllos IPK GER	2629	67411	66227	7013	0.0397
FB22_003 T. platyphyllos IPK GER	2376	63355	62161	6623	0.0382
FB22_011 T. platyphyllos Herzberg GER	1943	58524	57311	6110	0.0339
FB22_025 T. platyphyllos Jura FRA	2384	67224	66142	6969	0.0360
FB22_008 T. x euchlora IPK	4341	64133	62902	6780	0.0690
FB23_001 T. dasystyla BG Munich	3721	56517	55209	6030	0.0674
T01 T. dasy. sabe. HC IRN	3500	60581	59052	6427	0.0593
T02 T. dasy. cauc. HC IRN	3825	64218	62715	6852	0.0610
T03 T. dasy. cauc. HC IRN	4112	65948	64496	7023	0.0638
T04 T. dasy. sabe. HC IRN	4133	67238	65994	7165	0.0626
T05 T. dasy. cauc. HC IRN	4119	66251	64793	7027	0.0636
T06 T. dasy. cauc. HC IRN	3783	64235	62473	6826	0.0606
T07 T. dasy. cauc. HC IRN	4178	67175	65884	7158	0.0634
T08 T. dasy. cauc. HC IRN	4132	66766	65514	7107	0.0631
T09 T. dasy. cauc. HC IRN	4092	65349	63827	6971	0.0641
T10 T. dasy. cauc. HC IRN	4005	65993	64507	7041	0.0621
T11 T. dasy. cauc. HC IRN	4160	66716	65244	7109	0.0638
T12 T. dasy. cauc. HC IRN	3721	64565	62962	6876	0.0591
T13 T. dasy. sabe. HC IRN	3980	65202	63614	6942	0.0626
T14 T. dasy. cauc. HC IRN	4289	67268	65948	7151	0.0650
T15 T. dasy. sabe. HC IRN	4211	67326	66094	7164	0.0637
T16 T. dasy. cauc. HW IRN	4120	64781	63145	6910	0.0652
T17 T. dasy. cauc. HW IRN	4015	65832	64480	7039	0.0623
T18 T. dasy. cauc. HW IRN	3989	64769	63283	6906	0.0630
T19 T. dasy. cauc. HW IRN	4210	65472	64097	6990	0.0657
T20 T. dasy. cauc. HW IRN	4123	65308	63918	6971	0.0645
T21 T. dasy. cauc. HW IRN	4152	66061	64891	7043	0.0640
T22 T. dasy. cauc. HW IRN	4430	66232	65052	7089	0.0681
T23 T. dasy. cauc. HW IRN	4245	65745	64595	7056	0.0657
T24 T. dasy. cauc. HW IRN	4189	65797	64597	7001	0.0648
T25 T. dasy. cauc. HW IRN	4112	65948	64496	7022	0.0638
T26 T. dasy. cauc. HW IRN	4281	66102	64829	7049	0.0660
T27 T. dasy. cauc. HW IRN	4226	66130	64929	7040	0.0651
T28 T. dasy. cauc. HW IRN	3836	62801	61234	6707	0.0626
T29 T. dasy. cauc. HW IRN	4317	65531	64149	6995	0.0673
T30 T. dasy. cauc. HC IRN	4132	67103	65704	7133	0.0629
T31 T. dasy. cauc. HC IRN	4011	66758	65372	7111	0.0614
T32 T. dasy. cauc. HC IRN	3845	66744	65110	7086	0.0591

Individual	Observed het. positions	Sites	NetSites	NLoci	Fraction of heterozygous sites
T33 T. dasy. cauc. HC IRN	3915	65930	64533	7022	0.0607
T34 T. dasy. cauc. HC IRN	3800	66116	64688	7045	0.0587
T35 T. dasy. cauc. HC IRN	3938	66633	65142	7086	0.0605
T36 T. dasy. cauc. HC IRN	4124	66526	65238	7097	0.0632
T37 T. dasy. cauc. HC IRN	4030	65673	64167	7007	0.0628
T38 T. dasy. cauc. HC IRN	4076	66772	65534	7126	0.0622
T39 T. dasy. sabe. HC IRN	4154	66557	65173	7107	0.0637
T40 T. dasy. sabe. HC IRN	4046	66377	64992	7070	0.0623
T41 T. dasy. cauc. HE IRN	3665	64445	62959	6862	0.0582
T42 T. dasy. cauc. HE IRN	3812	65996	64438	7012	0.0592
T43 T. dasy. cauc. HE IRN	3334	62586	61019	6673	0.0546
T44 T. dasy. cauc. HE IRN	3278	60402	58767	6435	0.0558
T45 T. dasy. cauc. HE IRN	3292	62140	60547	6636	0.0544
T46 T. dasy. cauc. HE IRN	3910	66655	65200	7105	0.0600
T47 T. dasy. cauc. HE IRN	3922	65942	64558	7036	0.0608
T48 T. dasy. cauc. HE IRN	2408	45757	44453	4814	0.0542
T49 T. dasy. cauc. HE IRN	3909	66698	65179	7103	0.0600
T50 T. dasy. cauc. HE IRN	4095	66521	65100	7102	0.0629
T51 T. dasy. cauc. HE IRN	3982	66345	65001	7069	0.0613
T52 T. dasy. cauc. HE IRN	4057	67597	66132	7191	0.0613
T53 T. dasy. cauc. HE IRN	4112	67460	66126	7174	0.0622
T54 T. dasy. cauc. HE IRN	4019	67458	66119	7202	0.0608
T55 T. dasy. cauc. HE IRN	4119	64605	62868	6913	0.0655
T56 T. dasy. cauc. HE IRN	4017	66985	65617	7127	0.0612
T57 T. dasy. cauc. HE IRN	4121	66645	65116	7084	0.0633
T58 T. dasy. cauc. HE IRN	4232	67834	66666	7209	0.0635
T59 T. dasy. sabe. HC IRN	4279	67100	65733	7145	0.0651
T60 T. dasy. cauc. HC IRN	4094	67491	66223	7170	0.0618
T61 T. dasy. cauc. HC IRN	3393	62908	61366	6694	0.0553
T62 T. dasy. sabe. HC IRN	3917	67231	65866	7135	0.0595
T63 T. dasy. cauc. HC IRN	3985	66940	65662	7131	0.0607
T64 T. dasy. cauc. HC IRN	4129	67293	66063	7163	0.0625
T65 T. dasy. sabe. HC IRN	4236	66574	65239	7102	0.0649
T66 T. dasy. sabe. HC IRN	4197	67511	66301	7186	0.0633
T67 T. dasy. cauc. HE IRN	4169	67144	65859	7158	0.0633
T68 T. dasy. cauc. HE IRN	4002	67019	65641	7125	0.0610
T69 T. dasy. cauc. HE IRN	4126	67704	66314	7194	0.0622
T70 T. dasy. cauc. HE IRN	4069	67278	65971	7144	0.0617
T71 T. dasy. cauc. HE IRN	4162	67532	66289	7196	0.0628
T72 T. dasy. cauc. HE IRN	4083	67116	65829	7119	0.0620

Individual	Observed het. positions	Sites	NetSites	NLoci	Fraction of heterozygous sites
T73 T. dasy. sabe. HC IRN	4289	66553	65283	7110	0.0657
T74 T. dasy. sabe. HC IRN	4252	66801	65482	7122	0.0649
T75 T. dasy. sabe. HC IRN	4122	66527	65163	7075	0.0633
T76 T. dasy. sabe. HC IRN	4117	65823	64187	7037	0.0641
T77 T. dasy. cauc. HC IRN	4041	67102	65877	7144	0.0613
T78 T. dasy. sabe. HC IRN	4220	67531	66311	7188	0.0636
T79 T. dasy. cauc. HC IRN	4143	67925	66721	7219	0.0621
T80 T. dasy. cauc. HC IRN	4192	67545	66436	7193	0.0631
T81 T. dasy. cauc. HC IRN	4200	67318	66089	7178	0.0636
T82 T. dasy. sabe. HC IRN	4163	67663	66373	7181	0.0627
T83 T. dasy. sabe. HC IRN	4303	67044	65927	7149	0.0653
T84 T. dasy. sabe. HC IRN	3988	67391	66154	7178	0.0603
T85 T. dasy. sabe. HC IRN	4282	67141	65850	7156	0.0650
